# Supplementary material for: Interventions Targeting Bottle and Formula Feeding in the Prevention and Treatment of Early Childhood Caries, Overweight and Obesity: An Integrative Review
Source: Int J Environ Res Public Health. 2021 Nov 23;18(23):12304. doi: 10.3390/ijerph182312304 (PMC8656950; doi:10.3390/ijerph182312304)
Supplement: Supplementary file 1 [file ijerph-18-12304-s001.zip › Suppt File 2. Eligibility criteria.pdf]

Supplementary Table 2. Search strategy eligibility criteria.

**Criterion and definition**	**Included**	**Excluded**
Population or problem: characteristics of population; or problem, condition or disease of interest	Parents and caregivers of:<br>• Infants and young children fed with infant formula<br>• Infants and young children fed using a feeding bottle	Parents and caregivers of:<br>• Infants and young children not being fed infant formula<br>• Infants and young children not fed using a feeding bottle<br>• Toddlers and young child cohorts without children aged ≤2 years
Interest: defined event, activity, experience or process	Health outcomes that include:<br>• Dental caries or ECC prevalence<br>• Infant or baby anthropometry<br><br>Behaviour outcomes that include:<br>• Infant or baby bottle use<br>• Infant or baby feeding practices<br>• Cup use<br>• Dietary dental caries-related behaviour<br>• Parental or caregiver knowledge or awareness of best-practice behaviours<br>• Interventionist behaviour in delivering education for best-practice behaviours	• Subjective outcomes, e.g. participant feedback, health professional attitudes, parent attitudes, satisfaction with intervention.
Context: setting or distinct characteristics	Interventions that address:<br>• Improved formula feeding practices, e.g. responsive feeding, avoiding overfeeding<br>• Dietary dental caries-preventative behaviours, e.g. avoidance of sugar-sweetened drinks or juice, use of water, timing of cariogenic food and beverage intake<br>• Infant or baby bottle cessation<br>• Transition from bottle to cup use	• Multi-faceted intervention, where improving formula feeding practices, dental caries-preventative behaviour, and bottle cessation or transition to cup use are not the primary focus of the intervention
Other	• Published in English<br>• Any publication date	• Non-English language<br>• Study terminated before completion<br>• Full text not available
