# Supplementary material for: Interventions Targeting Bottle and Formula Feeding in the Prevention and Treatment of Early Childhood Caries, Overweight and Obesity: An Integrative Review
Source: Int J Environ Res Public Health. 2021 Nov 23;18(23):12304. doi: 10.3390/ijerph182312304 (PMC8656950; doi:10.3390/ijerph182312304)
Supplement: Supplementary file 1 [file ijerph-18-12304-s001.zip › Suppt File 3. CINAHL search strategy example.pdf]

Supplementary Table 3. Search strategy utilising the Population/Problem, Interest, Context framework, used in the CINAHL database.

Search terms	Formula or bottle feeding	Parent or caregiver education	Intervention	Formula or bottle cessation	Dental caries
	(MH "Infant Feeding, Supplemental") OR (MH "Infant Formula") OR (MH "Bottle Feeding")	(MH "Patient Education+") OR (MH "Patient Discharge Education")	AB (program or intervention or training or treatment or education)	TX (stop* OR quit* OR cessation OR cease OR wean) N3 (bottle* OR formula)	TX tooth decay or caries or dental caries or decayed tooth
	TX infant formula or formula feed* or formula milk	(MH "Parenting Education")	TI (program or intervention or training or treatment or education)		(MH "Dental Care+")
	TX bottle feed*	(MH "Support, Psychosocial+")	AB (trial or study or rct or experiment)		(MH "Dental Caries")
	TX formula feed*	(MH "Childbirth Education")	TI (trial or study or rct or experiment)		
	TX bottle use*	(MH "Learning Environment+")			
	TX baby milk feed*				
	TX baby bottle				
	TX mix* feed*				
	TX milk powder or powder* milk				
**Search strategy framework utilising combinations of search terms**					
**Interventions on education about infant formula or bottle use**	✓ (Problem/Interest)	✓ (Context)	✓ (Context)		
**Infant formula or bottle cessation**	✓ (Problem)			✓ (Interest)	
**Interventions about infant formula or bottle use and dental caries**	✓ (Problem/Interest)		✓ (Context)		✓ (Problem/Interest)
