# Supplementary material for: Interventions Targeting Bottle and Formula Feeding in the Prevention and Treatment of Early Childhood Caries, Overweight and Obesity: An Integrative Review
Source: Int J Environ Res Public Health. 2021 Nov 23;18(23):12304. doi: 10.3390/ijerph182312304 (PMC8656950; doi:10.3390/ijerph182312304)
Supplement: Supplementary file 1 [file ijerph-18-12304-s001.zip › Suppt File 4. Database search strategies.pdf]

Supplementary Tables 1: search strategies used for academic databases

<u>Information about the PICo used to inform the search strategy framework</u>

https://libguides.murdoch.edu.au/systematic/PICO

PICo for Qualitative Studies

P	I	Co
**Population or Problem**	**Interest**	**Context**
What are the characteristics of the Population or the patient?<br><br>What is the Problem, condition or disease you are interested in?	Interest relates to a defined event, activity, experience or process	Context is the setting or distinct characteristics

Here is an example of a clinical question that outlines the PICo components:

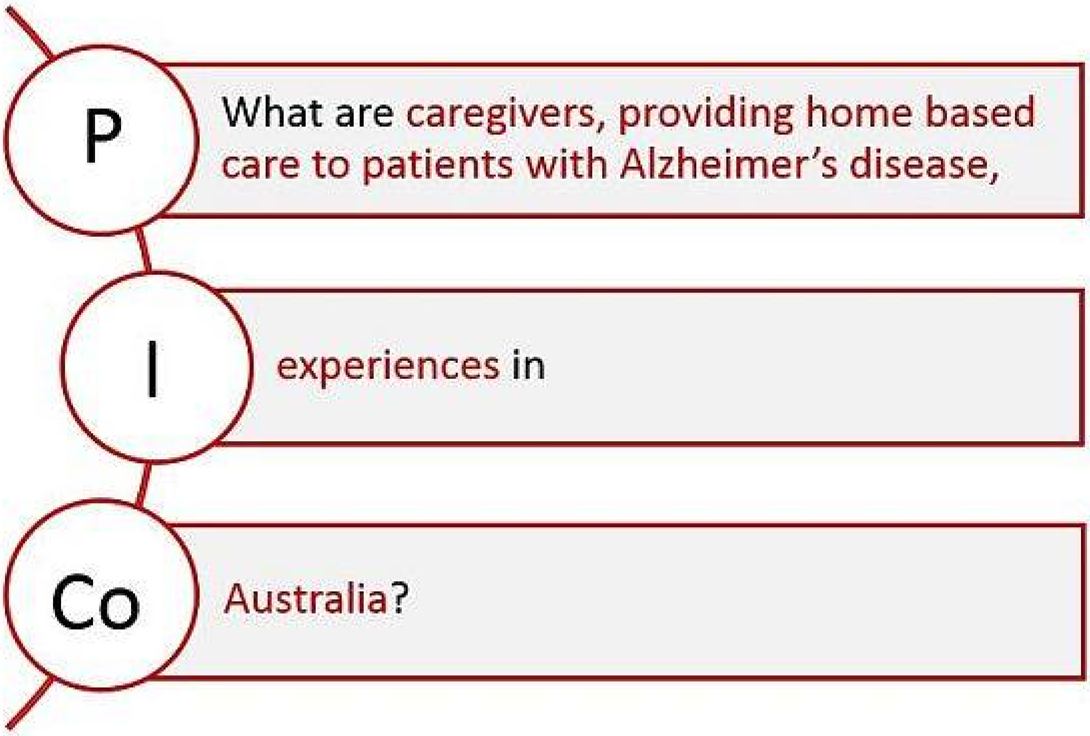

S18	S10 AND S16 AND S17	View Results (533)<br>View Details<br>Edit
S17	AB (program or intervention or training or treatment or education) OR TI (program or intervention or training or treatment or education) OR AB (trial or study or rct or experiment) OR TI (trial or study or rct or experiment)	View Results (2,840,346)<br>View Details<br>Edit
S16	S11 OR S12 OR S13 OR S14 OR S15	View Results (175,960)<br>View Details<br>Edit
S15	(MH "Learning Environment+")	View Results (12,085)<br>View Details<br>Edit
S14	(MH "Childbirth Education")	View Results (2,655)<br>View Details<br>Edit
S13	(MH "Support, Psychosocial+")	View Results (83,103)<br>View Details<br>Edit
S12	(MH "Parenting Education")	View Results (2,892)<br>View Details<br>Edit
S11	(MH "Patient Education+") OR (MH "Patient Discharge Education")	View Results (79,901)<br>View Details<br>Edit
S10	S1 OR S2 OR S3 OR S4 OR S5 OR S6 OR S7 OR S8 OR S9	View Results (20,387)<br>View Details<br>Edit
S9	TX milk powder or powder* milk	View Results (1,592)<br>View Details<br>Edit
S8	TX mix* feed*	View Results (1,651)<br>View Details<br>Edit
S7	TX infant formula or formula feed* or formula milk	View Results (9,604)<br>View Details<br>Edit
S6	TX baby milk feed*	View Results (511)<br>View Details<br>Edit
S5	TX bottle use*	View Results (3,358)<br>View Details<br>Edit
S4	TX formula feed*	View Results (4,277)<br>View Details<br>Edit
S3	TX bottle feed*	View Results (4,588)<br>View Details<br>Edit

S2	TX baby bottle	View Results (1,688)<br>View Details<br>Edit
S1	(MH "Infant Feeding, Supplemental") OR (MH "Infant Formula") OR (MH "Bottle Feeding")	View Results (5,916)<br>View Details<br>Edit

CINAHL, 31/7/20, search terms and key words for 'infant formula' + search terms for 'parent education' + key words on programs, studies and interventions

Search ID#	Search Terms	Actions
S12	S10 AND S11	View Results (209) View Details Edit
S11	TX (stop* OR quit* OR cessation OR cease OR wean) N3 (bottle* OR formula)	View Results (481) View Details Edit
S10	S1 OR S2 OR S3 OR S4 OR S5 OR S6 OR S7 OR S8 OR S9	View Results (20,387) View Details Edit
S9	TX milk powder or powder* milk	View Results (1,592) View Details Edit
S8	TX mix* feed*	View Results (1,651) View Details Edit
S7	TX infant formula or formula feed* or formula milk	View Results (9,604) View Details Edit
S6	TX baby milk feed*	View Results (511) View Details Edit
S5	TX bottle use*	View Results (3,358) View Details Edit
S4	TX formula feed*	View Results (4,277) View Details Edit
S3	TX bottle feed*	View Results (4,588) View Details Edit
S2	TX baby bottle	View Results (1,688) View Details Edit
S1	(MH "Infant Feeding, Supplemental") OR (MH "Infant Formula") OR (MH "Bottle Feeding")	View Results (5,916) View Details Edit

CINAHL, 31/7/20, search terms and key words for ‘infant formula’ + key words for ‘stopping’

S16	S10 AND S14 AND S15	View Results (517)<br>View Details<br>Edit
S15	AB (program or intervention or training or treatment or education) OR TI (program or intervention or training or treatment or education) OR AB (trial or study or rct or experiment) OR TI (trial or study or rct or experiment)	View Results (2,840,346)<br>View Details<br>Edit
S14	S11 OR S12 OR S13	View Results (59,610)<br>View Details<br>Edit
S13	TX tooth decay or caries or dental caries or decayed tooth	View Results (44,704)<br>View Details<br>Edit
S12	(MH "Dental Care+")	View Results (18,174)<br>View Details<br>Edit
S11	(MH "Dental Caries")	View Results (12,312)<br>View Details<br>Edit
S10	S1 OR S2 OR S3 OR S4 OR S5 OR S6 OR S7 OR S8 OR S9	View Results (20,387)<br>View Details<br>Edit
S9	TX milk powder or powder* milk	View Results (1,592)<br>View Details<br>Edit
S8	TX mix* feed*	View Results (1,651)<br>View Details<br>Edit
S7	TX infant formula or formula feed* or formula milk	View Results (9,604)<br>View Details<br>Edit
S6	TX baby milk feed*	View Results (511)<br>View Details<br>Edit
S5	TX bottle use*	View Results (3,358)<br>View Details<br>Edit
S4	TX formula feed*	View Results (4,277)<br>View Details<br>Edit
S3	TX bottle feed*	View Results (4,588)<br>View Details

		Edit
S2	TX baby bottle	View Results (1,688)<br>View Details<br>Edit
S1	(MH "Infant Feeding, Supplemental") OR (MH "Infant Formula") OR (MH "Bottle Feeding")	View Results (5,916)<br>View Details<br>Edit

CINAHL, 31/7/20, search terms and key words for ‘infant formula’ + search terms for ‘dental caries’ + key words on programs, studies and interventions

# ▲	Searches	Results
1	Infant Formula/	4537
2	Bottle Feeding/	3808
3	bottle feed*.mp.	4902
4	formula feed*.mp.	1914
5	bottle use*.mp.	126
6	baby milk feed*.mp.	2
7	infant formula feed*.mp.	92
8	baby formula feed*.mp.	2
9	((formula or milk powder or powdered milk) adj3 (infant* or bab* or toddler*)).mp.	8814
10	1 or 2 or 3 or 4 or 5 or 6 or 7 or 8 or 9	14097
11	Parenting/	16717
12	Patient Education as Topic/	85150
13	Social Support/ or Self-Help Groups/	77668
14	Prenatal Care/	27669
15	11 or 12 or 13 or 14	199314
16	Randomized Controlled Trial/ or Clinical Trial/ or trial.mp.	1196881
17	study.mp.	9144217
18	program.mp.	503569
19	intervention.mp.	622025
20	treatment.mp.	4867044
21	experiment.mp.	318324
22	16 or 17 or 18 or 19 or 20 or 21	12694744
23	10 and 15 and 22	280

Medline, 3/8/20, search terms and key words for 'infant formula' + search terms for 'parent education' + search terms and key words on programs, studies and interventions

# ▲	Searches	Results
1	Infant Formula/	4547
2	Bottle Feeding/	3811
3	bottle feed*.mp.	4906
4	formula feed*.mp.	1915
5	bottle use*.mp.	126
6	baby milk feed*.mp.	2
7	infant formula feed*.mp.	92
8	baby formula feed*.mp.	2
9	((formula or milk powder or powdered milk) adj3 (infant* or bab* or toddler*)).mp.	8827
10	1 or 2 or 3 or 4 or 5 or 6 or 7 or 8 or 9	14113
11	((stop* or quit* or cessation or cease or wean) adj3 (bottle* or formula)).mp. [mp=title, abstract, original title, name of substance word, subject heading word, floating sub-heading word, keyword heading word, organism supplementary concept word, protocol supplementary concept word, rare disease supplementary concept word, unique identifier, synonyms]	144
12	10 and 11	37

Medline, 5/8/20, search terms and key words for ‘infant formula’ + key words for ‘stopping’

# ▲	Searches	Results
1	Infant Formula/	4547
2	Bottle Feeding/	3811
3	bottle feed*.mp.	4906
4	formula feed*.mp.	1915
5	bottle use*.mp.	126
6	baby milk feed*.mp.	2
7	infant formula feed*.mp.	92
8	baby formula feed*.mp.	2
9	((formula or milk powder or powdered milk) adj3 (infant* or bab* or toddler*)).mp.	8827
10	1 or 2 or 3 or 4 or 5 or 6 or 7 or 8 or 9	14113
11	dental caries.mp. or Dental Caries/	51279
12	dental care.mp. or Dental Care/	40012
13	tooth decay.mp.	1290
14	11 or 12 or 13	86159
15	Randomized Controlled Trial/ or Clinical Trial/ or trial.mp.	1198303
16	study.mp.	9156581
17	program.mp.	504279
18	intervention.mp.	623261
19	treatment.mp.	4873742
20	experiment.mp.	318638
21	15 or 16 or 17 or 18 or 19 or 20	12711425
22	10 and 14 and 21	347

Medline, 5/8/20, search terms and key words for ‘infant formula’ + search terms for ‘dental caries’ + key words on programs, studies and interventions

# ▲	Searches	Results
1	infant formula.mp. or artificial milk/	15769
2	bottle feeding/	4102
3	bottle feed*.mp.	4875
4	formula feed*.mp.	2650
5	bottle use*.mp.	155
6	baby milk feed*.mp.	1
7	infant formula feed*.mp.	115
8	baby formula feed*.mp.	2
9	((formula or milk powder or powdered milk) adj3 (infant* or bab* or toddler*)).mp. [mp=title, abstract, heading word, drug trade name, original title, device manufacturer, drug manufacturer, device trade name, keyword, floating subheading word, candidate term word]	8217
10	1 or 2 or 3 or 4 or 5 or 6 or 7 or 8 or 9	22059
11	support group/ or social support/	102485
12	education/ or health education/ or parent education.mp. or education program/	551797
13	parenting education/	1271
14	parenting.mp. or child parent relation/	60550
15	prenatal care/	39939
16	11 or 12 or 13 or 14 or 15	729630
17	trial.mp. or randomized controlled trial/ or community trial/ or clinical trial/ or controlled clinical trial/	2161614
18	program.mp. or health program/ or community program/ or education program/	948948

19	study/ or clinical study/	170546
20	intervention.mp. or nursing intervention/ or intervention study/	980254
21	treatment.mp.	7214545
22	experiment.mp. or experiment/	3355921
23	17 or 18 or 19 or 20 or 21 or 22	11983095
24	10 and 16 and 23	447

Embase, 5/8/20, search terms and key words for 'infant formula' + search terms for 'parent education' + search terms and key words on programs, studies and interventions

# ▲	Searches	Results
1	infant formula.mp. or artificial milk/	15769
2	bottle feeding/	4102
3	bottle feed*.mp.	4875
4	formula feed*.mp.	2650
5	bottle use*.mp.	155
6	baby milk feed*.mp.	1
7	infant formula feed*.mp.	115
8	baby formula feed*.mp.	2
9	((formula or milk powder or powdered milk) adj3 (infant* or bab* or toddler*)).mp. [mp=title, abstract, heading word, drug trade name, original title, device manufacturer, drug manufacturer, device trade name, keyword, floating subheading word, candidate term word]	8217
10	1 or 2 or 3 or 4 or 5 or 6 or 7 or 8 or 9	22059
11	((stop* or quit* or cessation or cease or wean) adj3 (bottle* or formula)).mp. [mp=title, abstract, heading word, drug trade name, original title, device manufacturer, drug manufacturer, device trade name, keyword, floating subheading word, candidate term word]	207
12	10 and 11	45

Embase, 5/8/20, search terms and key words for ‘infant formula’ + key words for ‘stopping’

# ▲	Searches	Results
1	infant formula.mp. or artificial milk/	15769
2	bottle feeding/	4102
3	bottle feed*.mp.	4875
4	formula feed*.mp.	2650
5	bottle use*.mp.	155
6	baby milk feed*.mp.	1
7	infant formula feed*.mp.	115
8	baby formula feed*.mp.	2
9	((formula or milk powder or powdered milk) adj3 (infant* or bab* or toddler*)).mp. [mp=title, abstract, heading word, drug trade name, original title, device manufacturer, drug manufacturer, device trade name, keyword, floating subheading word, candidate term word]	8217
10	1 or 2 or 3 or 4 or 5 or 6 or 7 or 8 or 9	22059
11	trial.mp. or randomized controlled trial/ or community trial/ or clinical trial/ or controlled clinical trial/	2161614
12	program.mp. or health program/ or community program/ or education program/	948948
13	study/ or clinical study/	170546
14	intervention.mp. or nursing intervention/ or intervention study/	980254
15	treatment.mp.	7214545
16	experiment.mp. or experiment/	3355921
17	11 or 12 or 13 or 14 or 15 or 16	11983095
18	dental caries.mp. or dental caries/	53901
19	dental care.mp. or dental procedure/	82211

20	tooth decay.mp.	1561
21	18 or 19 or 20	127515
22	10 and 17 and 21	154

Embase, 5/8/20, search terms and key words for ‘infant formula’ + search terms and key words for ‘dental caries’ + search terms and key words on programs, studies and interventions

# ▲	Searches	Results
1	infant formula.mp. or infant formulae/	10939
2	bottle feeding.mp. or bottle feeding/	1471
3	bottle feed*.mp.	1598
4	formula feed*.mp.	1259
5	bottle use*.mp.	67
6	baby milk feed*.mp.	1
7	infant formula feed*.mp.	82
8	baby formula feed*.mp.	1
9	((formula or milk powder or powdered milk) adj3 (infant* or bab* or toddler*)).mp. [mp=abstract, title, original title, broad terms, heading words, identifiers, cabicodes]	11207
10	1 or 2 or 3 or 4 or 5 or 6 or 7 or 8 or 9	13554
11	patient education/ or nutrition education.sh. or counselling.sh. or patient education.mp.	19552
12	health services.sh. or parent education/ or parent education.mp.	82658
13	parenting class.mp.	3
14	support systems.sh.	3739
15	social support.mp.	8185
16	parent support.mp.	78
17	11 or 12 or 13 or 14 or 15 or 16	108359
18	trial.mp. or clinical trials/ or trials/ or randomized controlled trials/	128133
19	program.mp. or programs/ or "workshops (programs)"/	77554
20	study.mp. or research/	1550898
21	intervention/ or intervention.mp.	118026
22	treatment/ or treatment.mp.	748233
23	experiment.mp. or experiments/	72288
24	18 or 19 or 20 or 21 or 22 or 23	2113644
25	10 and 17 and 24	173

Global Health, 6/8/20, search terms and key words for ‘infant formula’ + search terms and key words for ‘parent education’ + search terms and key words on programs, studies and interventions

# ▲	Searches	Results
1	infant formula.mp. or infant formulae/	10939
2	bottle feeding.mp. or bottle feeding/	1471
3	bottle feed*.mp.	1598
4	formula feed*.mp.	1259
5	bottle use*.mp.	67
6	baby milk feed*.mp.	1
7	infant formula feed*.mp.	82
8	baby formula feed*.mp.	1
9	((formula or milk powder or powdered milk) adj3 (infant* or bab* or toddler*)).mp. [mp=abstract, title, original title, broad terms, heading words, identifiers, cabicodes]	11207
10	1 or 2 or 3 or 4 or 5 or 6 or 7 or 8 or 9	13554
11	trial.mp. or clinical trials/ or trials/ or randomized controlled trials/	128133
12	program.mp. or programs/ or "workshops (programs)"/	77554
13	study.mp. or research/	1550898
14	intervention/ or intervention.mp.	118026
15	treatment/ or treatment.mp.	748233
16	experiment.mp. or experiments/	72288
17	11 or 12 or 13 or 14 or 15 or 16	2113644
18	dental caries.mp. or dental caries/	11287
19	dental care.mp. or dental health.sh.	8618
20	tooth decay.mp.	10634
21	18 or 19 or 20	17424
22	10 and 17 and 21	96

Global Health, 6/8/20, search terms and key words for ‘infant formula’ + search terms and key words for ‘dental caries’ + search terms and key words on programs, studies and interventions

# ▲	**Searches**	Results
1	infant formula.mp. or infant formulae/	10939
2	bottle feeding.mp. or bottle feeding/	1471
3	bottle feed*.mp.	1598
4	formula feed*.mp.	1259
5	bottle use*.mp.	67
6	baby milk feed*.mp.	1
7	infant formula feed*.mp.	82
8	baby formula feed*.mp.	1
9	((formula or milk powder or powdered milk) adj3 (infant* or bab* or toddler*)).mp. [mp=abstract, title, original title, broad terms, heading words, identifiers, cabicodes]	11207
10	1 or 2 or 3 or 4 or 5 or 6 or 7 or 8 or 9	13554
11	((stop* or quit* or cessation or cease or wean) adj3 (bottle* or formula)).mp. [mp=abstract, title, original title, broad terms, heading words, identifiers, cabicodes]	205
12	10 and 11	19

Global Health, 6/8/20, search terms and key words for ‘infant formula’ + key words for ‘stopping’

16	((TITLE-ABS-KEY (infant AND formula)) OR (TITLE-ABS-KEY (bottle AND feeding)) OR (TITLE-ABS-KEY (formula AND feeding)) OR (TITLE-ABS-KEY (bottle AND feed*)) OR (TITLE-ABS-KEY (formula AND feed*)) OR (TITLE-ABS-KEY (bottle AND use*)) OR (TITLE-ABS-KEY (baby AND milk AND feed*)) OR (TITLE-ABS-KEY (infant AND formula AND feed*)) OR (TITLE-ABS-KEY (baby AND formula AND feed*)) OR (TITLE-ABS-KEY (formula) OR TITLE-ABS-KEY (milk AND powder) OR TITLE-ABS-KEY (powdered AND milk) W/3 TITLE-ABS-KEY (infant*) OR TITLE-ABS-KEY (bab*) OR TITLE-ABS-KEY (toddler*))) AND ((TITLE-ABS-KEY (parent* AND education) OR TITLE-ABS-KEY (parent* AND class) OR TITLE-ABS-KEY (patient AND education) OR TITLE-ABS-KEY (parent* AND support*) OR TITLE-ABS-KEY (parent* AND advice*) OR TITLE-ABS-KEY (prenatal AND class) OR TITLE-ABS-KEY (antenatal AND class) OR TITLE-ABS-KEY (social AND support))) AND ((TITLE-ABS-KEY (trial) OR TITLE-ABS-KEY (random* AND control* AND trial) OR TITLE-ABS-KEY (study) OR TITLE-ABS-KEY (program) OR TITLE-ABS-KEY (intervention) OR TITLE-ABS-KEY (study) OR TITLE-ABS-KEY (treatment) OR TITLE-ABS-KEY (experiment))) View Less	3,170 document results
15	(TITLE-ABS-KEY (trial) OR TITLE-ABS-KEY (random* AND control* AND trial) OR TITLE-ABS-KEY (study) OR TITLE-ABS-KEY (program) OR TITLE-ABS-KEY (intervention) OR TITLE-ABS-KEY (study) OR TITLE-ABS-KEY (treatment) OR TITLE-ABS-KEY (experiment))	34,180,723 document results
14	(TITLE-ABS-KEY (parent* AND education) OR TITLE-ABS-KEY (parent* AND class) OR TITLE-ABS-KEY (patient AND education) OR TITLE-ABS-KEY (parent* AND support*) OR TITLE-ABS-KEY (parent* AND advice*) OR TITLE-ABS-KEY (prenatal AND class) OR TITLE-ABS-KEY (antenatal AND class) OR TITLE-ABS-KEY (social AND support))	958,244 document results
13	(TITLE-ABS-KEY (infant AND formula)) OR (TITLE-ABS-KEY (bottle AND feeding)) OR (TITLE-ABS-KEY (formula AND feeding)) OR (TITLE-ABS-KEY (bottle AND feed*)) OR (TITLE-ABS-KEY (formula AND feed*)) OR (TITLE-ABS-KEY (bottle AND use*)) OR (TITLE-ABS-KEY (baby AND milk AND feed*)) OR (TITLE-ABS-KEY (infant AND formula AND feed*)) OR (TITLE-ABS-KEY (baby AND formula AND feed*)) OR (TITLE-ABS-KEY (formula) OR TITLE-ABS-KEY (milk AND powder) OR TITLE-ABS-KEY (powdered AND milk) W/3 TITLE-ABS-KEY (infant*) OR TITLE-ABS-KEY (bab*) OR TITLE-ABS-KEY (toddler*)) View Less	101,025 document results
12	TITLE-ABS-KEY (formula) OR TITLE-ABS-KEY (milk AND powder) OR TITLE-ABS-KEY-	54,028 document results

	KEY (powdered AND milk) W/3 TITLE-ABS-KEY (infant*) OR TITLE-ABS-KEY (bab*) OR TITLE-ABS-KEY (toddler*)	
9	TITLE-ABS-KEY (baby AND formula AND feed*)	2,241 document results
8	TITLE-ABS-KEY (infant AND formula AND feed*)	10,229 document results
7	TITLE-ABS-KEY (baby AND milk AND feed*)	4,926 document results
6	TITLE-ABS-KEY (bottle AND use*)	23,649 document results
5	TITLE-ABS-KEY (formula AND feed*)	21,297 document results
4	TITLE-ABS-KEY (bottle AND feed*)	8,904 document results
3	TITLE-ABS-KEY (formula AND feeding)	14,062 document results
2	TITLE-ABS-KEY (bottle AND feeding)	7,938 document results
1	TITLE-ABS-KEY (infant AND formula)	20,707 document results

Scopus, 6/8/20, key words for 'infant formula' + key words for 'parent education' + key words on programs, studies and interventions

History Count	Search Terms	Results
7	((TITLE-ABS-KEY (infant AND formula)) OR (TITLE-ABS-KEY (bottle AND feeding)) OR (TITLE-ABS-KEY (formula AND feeding)) OR (TITLE-ABS-KEY (bottle AND feed*)) OR (TITLE-ABS-KEY (formula AND feed*)) OR (TITLE-ABS-KEY (bottle AND use*)) OR (TITLE-ABS-KEY (baby AND milk AND feed*)) OR (TITLE-ABS-KEY (infant AND formula AND feed*)) OR (TITLE-ABS-KEY (baby AND formula AND feed*)) OR (TITLE-ABS-KEY (formula) OR TITLE-ABS-KEY (milk AND powder) OR TITLE-ABS-KEY (powdered AND milk) W/3 TITLE-ABS-KEY (infant*) OR TITLE-ABS-KEY (bab*) OR TITLE-ABS-KEY (toddler*))) AND ((TITLE-ABS-KEY (dental AND caries)) OR (TITLE-ABS-KEY (dental AND care)) OR (TITLE-ABS-KEY (tooth AND decay))) AND ((TITLE-ABS-KEY (trial) OR TITLE-ABS-KEY (random* AND control* AND trial) OR TITLE-ABS-KEY (study) OR TITLE-ABS-KEY (program) OR TITLE-ABS-KEY (intervention) OR TITLE-ABS-KEY (study) OR TITLE-ABS-KEY (treatment) OR TITLE-ABS-KEY (experiment))) View Less	894 document results
6	(TITLE-ABS-KEY (trial) OR TITLE-ABS-KEY (random* AND control* AND trial) OR TITLE-ABS-KEY (study) OR TITLE-ABS-KEY (program) OR TITLE-ABS-KEY (intervention) OR TITLE-ABS-KEY (study) OR TITLE-ABS-KEY (treatment) OR TITLE-ABS-KEY (experiment))	34,190,053 document results

History Count	Search Terms	Results
5	(TITLE-ABS-KEY (dental AND caries)) OR (TITLE-ABS-KEY (dental AND care)) OR (TITLE-ABS-KEY (tooth AND decay))	170,138 document results
4	TITLE-ABS-KEY (tooth AND decay)	3,900 document results
3	TITLE-ABS-KEY (dental AND care)	115,611 document results
2	TITLE-ABS-KEY (dental AND caries)	67,689 document results
1	(TITLE-ABS-KEY (infant AND formula)) OR (TITLE-ABS-KEY (bottle AND feeding)) OR (TITLE-ABS-KEY (formula AND feeding)) OR (TITLE-ABS-KEY (bottle AND feed*)) OR (TITLE-ABS-KEY (formula AND feed*)) OR (TITLE-ABS-KEY (bottle AND use*)) OR (TITLE-ABS-KEY (baby AND milk AND feed*)) OR (TITLE-ABS-KEY (infant AND formula AND feed*)) OR (TITLE-ABS-KEY (baby AND formula AND feed*)) OR (TITLE-ABS-KEY (formula) OR TITLE-ABS-KEY (milk AND powder) OR TITLE-ABS-KEY (powdered AND milk) W/3 TITLE-ABS-KEY (infant*) OR TITLE-ABS-KEY (bab*) OR TITLE-ABS-KEY (toddler*))	101,070 document results

Scopus, 7/8/20, key words for 'infant formula' + key words for 'dental caries' + key words on programs, studies and interventions

History Count	Search Terms	Results
3	((TITLE-ABS-KEY (infant AND formula)) OR (TITLE-ABS-KEY (bottle AND feeding)) OR (TITLE-ABS-KEY (formula AND feeding)) OR (TITLE-ABS-KEY (bottle AND feed*)) OR (TITLE-ABS-KEY (formula AND feed*)) OR (TITLE-ABS-KEY (bottle AND use*)) OR (TITLE-ABS-KEY (baby AND milk AND feed*)) OR (TITLE-ABS-KEY (infant AND formula AND feed*)) OR (TITLE-ABS-KEY (baby AND formula AND feed*)) OR (TITLE-ABS-KEY (formula) OR TITLE-ABS-KEY (milk AND powder) OR TITLE-ABS-KEY (powdered AND milk) W/3 TITLE-ABS-KEY (infant*) OR TITLE-ABS-KEY (bab*) OR TITLE-ABS-KEY (toddler*))) AND (ALL (stop* OR quit* OR cessation OR cease OR wean *) W/3 ALL (bottle* OR formula)) View Less	992 document results
2	ALL (stop* OR quit* OR cessation OR cease OR wean*) W/3 ALL (bottle* OR formula)	3,803 document results
1	(TITLE-ABS-KEY (infant AND formula)) OR (TITLE-ABS-KEY (bottle AND feeding)) OR (TITLE-ABS-KEY (formula AND feeding)) OR (TITLE-ABS-KEY (bottle AND feed*)) OR (TITLE-ABS-KEY (formula AND feed*)) OR (TITLE-ABS-KEY (bottle AND use*)) OR (TITLE-ABS-KEY (baby AND milk AND feed*)) OR (TITLE-ABS-KEY (infant AND formula AND feed*)) OR (TITLE-ABS-KEY (baby AND formula AND feed*)) OR (TITLE-ABS-KEY (formula) OR TITLE-ABS-KEY (milk AND powder) OR TITLE-ABS-KEY (powdered AND milk) W/3 TITLE-ABS-KEY (infant*) OR TITLE-ABS-KEY (bab*) OR TITLE-ABS-KEY (toddler*)) View Less	101,070 document results

Scopus, 7/8/20, key words for ‘infant formula’ + key words for ‘stopping’

Set	Search	Databases	Results
S33	((su(parenting class) OR ti(parenting class) OR ab(parenting class) OR mesh(parenting class)) OR (su(parent* class) OR ti(parent* class) OR ab(parent* class) OR mesh(parent* class)) OR (su(parent* education) OR ti(parent* education) OR ab(parent* education) OR mesh(parent* education)) OR (su(parent* support) OR ti(parent* support) OR ab(parent* support) OR mesh(parent* support)) OR (su(parent* advice*) OR ti(parent* advice*) OR ab(parent* advice*) OR mesh(parent* advice*)) OR (su(prenatal class) OR ti(prenatal class) OR ab(prenatal class) OR mesh(prenatal class)) OR (su(antenatal class) OR ti(antenatal class) OR ab(antenatal class) OR mesh(antenatal class)) OR (su(health education) OR ti(health education) OR ab(health education) OR mesh(health education)) OR (su(patient education) OR ti(patient education) OR ab(patient education) OR mesh(patient education)) OR (su(social support) OR ti(social support) OR ab(social support) OR mesh(social support))) AND ((su(trial) OR ab(trial) OR ti(trial) OR mesh(trial)) OR (su(study) OR ab(study) OR ti(study) OR mesh(study)) OR (su(program) OR ab(program) OR ti(program) OR mesh(program)) OR (su(intervention) OR ab(intervention) OR ti(intervention) OR mesh(intervention)) OR (su(treatment) OR ab(treatment) OR ti(treatment) OR mesh(treatment)) OR (su(experiment) OR ab(experiment) OR ti(experiment) OR mesh(experiment))) AND ((su(infant formula) OR ab(infant formula) OR ti(infant formula) OR issn(infant formula)) OR (su(infant formula) OR ab(infant formula) OR ti(infant formula) OR mesh(infant formula)) OR (su(formula feeding) OR ab(formula feeding) OR ti(formula feeding) OR mesh(formula feeding)) OR (su(bottle feeding) OR ab(bottle feeding) OR ti(bottle feeding) OR mesh(bottle feeding)) OR (su(bottle feed*) OR ab(bottle feed*) OR ti(bottle feed*) OR mesh(bottle feed*)) OR (su(formula feed*) OR ab(formula feed*) OR ti(formula feed*) OR mesh(formula feed*)) OR (su(bottle use*) OR ab(bottle use*) OR ti(bottle use*) OR mesh(bottle use*)) OR (su(baby milk feed*) OR ab(baby milk feed*) OR ti(baby milk feed*) OR mesh(baby milk feed*)) OR (su(baby formula feed*) OR ab(baby formula feed*) OR ti(baby formula feed*) OR mesh(baby formula feed*)) OR (su(infant formula feed*) OR ab(infant formula feed*) OR ti(infant formula feed*) OR mesh(infant formula feed*)))	Nursing & Allied Health Database	475
S32	(su(infant formula) OR ab(infant formula) OR ti(infant formula) OR mesh(infant formula)) OR (su(infant formula) OR ab(infant formula) OR ti(infant formula) OR	Nursing & Allied Health Database	6,954

Set	Search	Databases	Results
	mesh(infant formula)) OR (su(formula feeding) OR ab(formula feeding) OR ti(formula feeding) OR mesh(formula feeding)) OR (su(bottle feeding) OR ab(bottle feeding) OR ti(bottle feeding) OR mesh(bottle feeding)) OR (su(bottle feed*) OR ab(bottle feed*) OR ti(bottle feed*) OR mesh(bottle feed*)) OR (su(formula feed*) OR ab(formula feed*) OR ti(formula feed*) OR mesh(formula feed*)) OR (su(bottle use*) OR ab(bottle use*) OR ti(bottle use*) OR mesh(bottle use*)) OR (su(baby milk feed*) OR ab(baby milk feed*) OR ti(baby milk feed*) OR mesh(baby milk feed*)) OR (su(baby formula feed*) OR ab(baby formula feed*) OR ti(baby formula feed*) OR mesh(baby formula feed*)) OR (su(infant formula feed*) OR ab(infant formula feed*) OR ti(infant formula feed*) OR mesh(infant formula feed*))		
S31	(su(trial) OR ab(trial) OR ti(trial) OR mesh(trial)) OR (su(study) OR ab(study) OR ti(study) OR mesh(study)) OR (su(program) OR ab(program) OR ti(program) OR mesh(program)) OR (su(intervention) OR ab(intervention) OR ti(intervention) OR mesh(intervention)) OR (su(treatment) OR ab(treatment) OR ti(treatment) OR mesh(treatment)) OR (su(experiment) OR ab(experiment) OR ti(experiment) OR mesh(experiment))	Nursing & Allied Health Database	3,296,322
S30	su(experiment) OR ab(experiment) OR ti(experiment) OR mesh(experiment)	Nursing & Allied Health Database	108,903
S29	su(treatment) OR ab(treatment) OR ti(treatment) OR mesh(treatment)	Nursing & Allied Health Database	906,612
S28	su(intervention) OR ab(intervention) OR ti(intervention) OR mesh(intervention)	Nursing & Allied Health Database	277,612
S27	su(program) OR ab(program) OR ti(program) OR mesh(program)	Nursing & Allied Health Database	344,857
S26	su(study) OR ab(study) OR ti(study) OR mesh(study)	Nursing & Allied Health Database	2,481,696
S25	su(trial) OR ab(trial) OR ti(trial) OR mesh(trial)	Nursing & Allied Health Database	370,585

Set	Search	Databases	Results
S24	(su(parenting class) OR ti(parenting class) OR ab(parenting class) OR mesh(parenting class)) OR (su(parent* class) OR ti(parent* class) OR ab(parent* class) OR mesh(parent* class)) OR (su(parent* education) OR ti(parent* education) OR ab(parent* education) OR mesh(parent* education)) OR (su(parent* support) OR ti(parent* support) OR ab(parent* support) OR mesh(parent* support)) OR (su(parent* advice*) OR ti(parent* advice*) OR ab(parent* advice*) OR mesh(parent* advice*)) OR (su(prenatal class) OR ti(prenatal class) OR ab(prenatal class) OR mesh(prenatal class)) OR (su(antenatal class) OR ti(antenatal class) OR ab(antenatal class) OR mesh(antenatal class)) OR (su(health education) OR ti(health education) OR ab(health education) OR mesh(health education)) OR (su(patient education) OR ti(patient education) OR ab(patient education) OR mesh(patient education)) OR (su(social support) OR ti(social support) OR ab(social support) OR mesh(social support))	Nursing & Allied Health Database	217,064
S23	su(social support) OR ti(social support) OR ab(social support) OR mesh(social support)	Nursing & Allied Health Database	45,868
S22	su(patient education) OR ti(patient education) OR ab(patient education) OR mesh(patient education)	Nursing & Allied Health Database	63,564
S21	su(health education) OR ti(health education) OR ab(health education) OR mesh(health education)	Nursing & Allied Health Database	128,975
S20	su(antenatal class) OR ti(antenatal class) OR ab(antenatal class) OR mesh(antenatal class)	Nursing & Allied Health Database	198
S19	su(prenatal class) OR ti(prenatal class) OR ab(prenatal class) OR mesh(prenatal class)	Nursing & Allied Health Database	495
S18	su(parent* advice*) OR ti(parent* advice*) OR ab(parent* advice*) OR mesh(parent* advice*)	Nursing & Allied Health Database	1,470
S17	su(parent* support) OR ti(parent* support) OR ab(parent* support) OR mesh(parent* support)	Nursing & Allied Health Database	15,992

Set	Search	Databases	Results
S16	su(parent* education) OR ti(parent* education) OR ab(parent* education) OR mesh(parent* education)	Nursing & Allied Health Database	14,327
S15	su(parent* class) OR ti(parent* class) OR ab(parent* class) OR mesh(parent* class)	Nursing & Allied Health Database	2,872
S14	su(parenting class) OR ti(parenting class) OR ab(parenting class) OR mesh(parenting class)	Nursing & Allied Health Database	535
S10	su(infant formula feed*) OR ab(infant formula feed*) OR ti(infant formula feed*) OR mesh(infant formula feed*)	Nursing & Allied Health Database	1,392
S9	su(baby formula feed*) OR ab(baby formula feed*) OR ti(baby formula feed*) OR mesh(baby formula feed*)	Nursing & Allied Health Database	394
S8	su(baby milk feed*) OR ab(baby milk feed*) OR ti(baby milk feed*) OR mesh(baby milk feed*)	Nursing & Allied Health Database	865
S7	su(bottle use*) OR ab(bottle use*) OR ti(bottle use*) OR mesh(bottle use*)	Nursing & Allied Health Database	1,633
S6	su(formula feed*) OR ab(formula feed*) OR ti(formula feed*) OR mesh(formula feed*)	Nursing & Allied Health Database	2,203
S5	su(bottle feed*) OR ab(bottle feed*) OR ti(bottle feed*) OR mesh(bottle feed*)	Nursing & Allied Health Database	926
S4	su(bottle feeding) OR ab(bottle feeding) OR ti(bottle feeding) OR mesh(bottle feeding)	Nursing & Allied Health Database	834
S3	su(formula feeding) OR ab(formula feeding) OR ti(formula feeding) OR mesh(formula feeding)	Nursing & Allied Health Database	1,925
S2	su(infant formula) OR ab(infant formula) OR ti(infant formula) OR mesh(infant formula)	Nursing & Allied Health Database	3,598

Set	Search	Databases	Results
S1	su(infant formula) OR ab(infant formula) OR ti(infant formula) OR mesh(infant formula)	Nursing & Allied Health Database	3,598

ProQuest, 7/8/20, key words for ‘infant formula’ + key words for ‘parent education’ + key words on programs, studies and interventions

Set	Search	Databases	Results
S8	((su(trial) OR ab(trial) OR ti(trial) OR mesh(trial)) OR (su(study) OR ab(study) OR ti(study) OR mesh(study)) OR (su(program) OR ab(program) OR ti(program) OR mesh(program)) OR (su(intervention) OR ab(intervention) OR ti(intervention) OR mesh(intervention)) OR (su(treatment) OR ab(treatment) OR ti(treatment) OR mesh(treatment)) OR (su(experiment) OR ab(experiment) OR ti(experiment) OR mesh(experiment))) AND ((ab(dental caries) OR su(dental caries) OR ti(dental caries) OR mesh(dental caries)) OR (ab(dental care) OR su(dental care) OR ti(dental care) OR mesh(dental care)) OR (ab(tooth decay) OR su(tooth decay) OR ti(tooth decay) OR mesh(tooth decay))) AND ((su(infant formula) OR ab(infant formula) OR ti(infant formula) OR mesh(infant formula)) OR (su(infant formula) OR ab(infant formula) OR ti(infant formula) OR mesh(infant formula)) OR (su(formula feeding) OR ab(formula feeding) OR ti(formula feeding) OR mesh(formula feeding)) OR (su(bottle feeding) OR ab(bottle feeding) OR ti(bottle feeding) OR mesh(bottle feeding)) OR (su(bottle feed*) OR ab(bottle feed*) OR ti(bottle feed*) OR mesh(bottle feed*)) OR (su(formula feed*) OR ab(formula feed*) OR ti(formula feed*) OR mesh(formula feed*)) OR (su(bottle use*) OR ab(bottle use*) OR ti(bottle use*) OR mesh(bottle use*)) OR (su(baby milk feed*) OR ab(baby milk feed*) OR ti(baby milk feed*) OR mesh(baby milk feed*)) OR (su(baby formula feed*) OR ab(baby formula feed*) OR ti(baby formula feed*) OR mesh(baby formula feed*)) OR (su(infant formula feed*) OR ab(infant formula feed*) OR ti(infant formula feed*) OR mesh(infant formula feed*)))	Nursing & Allied Health Database	45
S7	(su(infant formula) OR ab(infant formula) OR ti(infant formula) OR mesh(infant formula)) OR (su(infant formula) OR ab(infant formula) OR ti(infant formula) OR mesh(infant formula)) OR (su(formula feeding) OR ab(formula feeding) OR ti(formula feeding) OR mesh(formula feeding)) OR (su(bottle feeding) OR ab(bottle feeding) OR ti(bottle feeding) OR mesh(bottle feeding)) OR (su(bottle feed*) OR ab(bottle feed*) OR ti(bottle feed*) OR mesh(bottle feed*)) OR (su(formula feed*) OR ab(formula feed*) OR ti(formula feed*) OR mesh(formula feed*)) OR (su(bottle use*) OR ab(bottle use*) OR ti(bottle use*) OR mesh(bottle use*)) OR (su(baby milk feed*) OR ab(baby milk feed*) OR ti(baby milk feed*) OR mesh(baby milk feed*)) OR (su(baby formula feed*) OR ab(baby formula feed*) OR ti(baby formula feed*) OR mesh(baby formula feed*)) OR (su(infant formula feed*) OR ab(infant formula feed*) OR ti(infant formula feed*) OR mesh(infant formula feed*))	Nursing & Allied Health Database	6,954

Set	Search	Databases	Results
S6	(ab(dental caries) OR su(dental caries) OR ti(dental caries) OR mesh(dental caries)) OR (ab(dental care) OR su(dental care) OR ti(dental care) OR mesh(dental care)) OR (ab(tooth decay) OR su(tooth decay) OR ti(tooth decay) OR mesh(tooth decay))	Nursing & Allied Health Database	13,355
S5	ab(tooth decay) OR su(tooth decay) OR ti(tooth decay) OR mesh(tooth decay)	Nursing & Allied Health Database	843
S4	ab(dental care) OR su(dental care) OR ti(dental care) OR mesh(dental care)	Nursing & Allied Health Database	10,221
S3	ab(dental caries) OR su(dental caries) OR ti(dental caries) OR mesh(dental caries)	Nursing & Allied Health Database	4,005
S2	(su(trial) OR ab(trial) OR ti(trial) OR mesh(trial)) OR (su(study) OR ab(study) OR ti(study) OR mesh(study)) OR (su(program) OR ab(program) OR ti(program) OR mesh(program)) OR (su(intervention) OR ab(intervention) OR ti(intervention) OR mesh(intervention)) OR (su(treatment) OR ab(treatment) OR ti(treatment) OR mesh(treatment)) OR (su(experiment) OR ab(experiment) OR ti(experiment) OR mesh(experiment))	Nursing & Allied Health Database	3,297,191

ProQuest, 10/8/20, key words for ‘infant formula’ + key words for ‘dental caries’ + key words on programs, studies and interventions

1	(Infant formulae or Infant formula).de. or infant formula.mp.	2645
2	Formula feeding.de. or formula feeding.mp.	680
3	Bottle feeding.de. or bottle feeding.mp.	1350
4	bottle feed*.mp. [mp=abstract, heading word, title]	1420
5	formula feed*.mp. [mp=abstract, heading word, title]	810
6	baby milk feed*.mp. [mp=abstract, heading word, title]	0
7	infant formula feed*.mp. [mp=abstract, heading word, title]	40
8	baby formula feed*.mp. [mp=abstract, heading word, title]	1
9	((formula or milk powder or powdered milk) adj3 (infant* or bab* or toddler*)).mp. [mp=abstract, heading word, title]	2310
10	1 or 2 or 3 or 4 or 5 or 6 or 7 or 8 or 9	4549
11	Education.de. or parent education.mp. or Social support.de.	5124
12	(Health education or Patient education or Maternal health services).de. or patient education.mp. or Antenatal care.de.	13593
13	Support.de.	32
14	Antenatal classes.de. or parenting class.mp.	12
15	parent support.mp.	76
16	Parents - education.de.	393
17	11 or 12 or 13 or 14 or 15 or 16	18596
18	(Research or Clinical trials or Randomised controlled trials).de. or trial.mp.	17742
19	Health promotion.de. or program.mp.	9740
20	study.de. or study.mp.	110647
21	intervention.mp.	15664
22	Treatment.de. or treatment.mp.	29557
23	experiment.mp. or Experiments.de.	409
24	18 or 19 or 20 or 21 or 22 or 23	143270
25	10 and 17 and 24	82

Maternity & Infant Care Database, 10/8/20, search terms and key words for ‘infant formula’ + search terms and key words for ‘parent education’ + search terms and key words on programs, studies and interventions

# ▲	Searches	Results
1	(Infant formulae or Infant formula).de. or infant formula.mp.	2645
2	Formula feeding.de. or formula feeding.mp.	680
3	Bottle feeding.de. or bottle feeding.mp.	1350
4	bottle feed*.mp. [mp=abstract, heading word, title]	1420
5	formula feed*.mp. [mp=abstract, heading word, title]	810
6	baby milk feed*.mp. [mp=abstract, heading word, title]	0
7	infant formula feed*.mp. [mp=abstract, heading word, title]	40
8	baby formula feed*.mp. [mp=abstract, heading word, title]	1
9	((formula or milk powder or powdered milk) adj3 (infant* or bab* or toddler*)).mp. [mp=abstract, heading word, title]	2310
10	1 or 2 or 3 or 4 or 5 or 6 or 7 or 8 or 9	4549
11	(Research or Clinical trials or Randomised controlled trials).de. or trial.mp.	17742
12	Health promotion.de. or program.mp.	9740
13	study.de. or study.mp.	110647
14	intervention.mp.	15664
15	Treatment.de. or treatment.mp.	29557
16	experiment.mp. or Experiments.de.	409
17	11 or 12 or 13 or 14 or 15 or 16	143270
18	(Dental caries or Dental care).de. or dental caries.mp. or Tooth diseases.de.	220
19	Dental care.de. or dental care.mp.	149
20	tooth decay.mp.	34
21	18 or 19 or 20	285
22	10 and 17 and 21	7

Maternity & Infant Care Database, 10/8/20, search terms and key words for ‘infant formula’ + search terms and key words for ‘dental caries’ + search terms and key words on programs, studies and interventions

1	(Infant formulae or Infant formula).de. or infant formula.mp.	2645
2	Formula feeding.de. or formula feeding.mp.	680
3	Bottle feeding.de. or bottle feeding.mp.	1350
4	bottle feed*.mp. [mp=abstract, heading word, title]	1420
5	formula feed*.mp. [mp=abstract, heading word, title]	810
6	baby milk feed*.mp. [mp=abstract, heading word, title]	0
7	infant formula feed*.mp. [mp=abstract, heading word, title]	40
8	baby formula feed*.mp. [mp=abstract, heading word, title]	1
9	((formula or milk powder or powdered milk) adj3 (infant* or bab* or toddler*)).mp. [mp=abstract, heading word, title]	2310
10	1 or 2 or 3 or 4 or 5 or 6 or 7 or 8 or 9	4549
11	((stop* or quit* or cessation or cease or wean) adj3 (bottle* or formula)).mp. [mp=abstract, heading word, title]	22
12	10 and 11	13

Maternity & Infant Care Database, 10/8/20, search terms and key words for ‘infant formula’ + key words for ‘stopping’

Search	Query	Results
#34	Search: ((((((((((((experiment[Title/Abstract]) OR (treatment[Title/Abstract])) OR (intervention[Title/Abstract])) OR (((Clinical Trial[MeSH Major Topic]) OR (Clinical Trial[MeSH Subheading])) OR (Clinical Trial[MeSH Terms]))) OR (study[Title/Abstract])) OR (Program[Title/Abstract])) OR (((Program[MeSH Major Topic]) OR (Program[MeSH Subheading])) OR (Program[MeSH Terms]))) OR (trial[Title/Abstract])) OR (((Pragmatic Clinical Trials as Topic[MeSH Major Topic]) OR (Pragmatic Clinical Trials as Topic[MeSH Subheading])) OR (Pragmatic Clinical Trials as Topic[MeSH Terms]))) OR (((Non-Randomized Controlled Trials as Topic[MeSH Major Topic]) OR (Non-Randomized Controlled Trials as Topic[MeSH Subheading])) OR (Non-Randomized Controlled Trials as Topic[MeSH Terms]))) OR (((Clinical Trials as Topic[MeSH Major Topic]) OR (Clinical Trials as Topic[MeSH Subheading])) OR (Clinical Trials as Topic[MeSH Terms]))) OR (((Randomized Controlled Trials as Topic[MeSH Major Topic]) OR (Randomized Controlled Trials as Topic[MeSH Subheading])) OR (Randomized Controlled Trials as Topic[MeSH Terms]))) AND ((((((((((Support Groups[MeSH Major Topic]) OR (Support Groups[MeSH Subheading])) OR (Support Groups[MeSH Terms])) OR (((Social Support[MeSH Major Topic]) OR (Social Support[MeSH Subheading])) OR (Social Support[MeSH Terms]))) OR (parent* support*[Title/Abstract])) OR (parent* class[Title/Abstract])) OR (((Patient Education Handout[MeSH Major Topic]) OR (Patient Education Handout[MeSH Subheading])) OR (Patient Education Handout[MeSH Terms]))) OR (((Patient Education as Topic[MeSH Major Topic]) OR (Patient Education as Topic[MeSH Subheading])) OR (Patient Education as Topic[MeSH Terms]))) OR (((Health Education[MeSH Major Topic]) OR (Health Education[MeSH Subheading])) OR (Health Education[MeSH Terms]))) OR (((Prenatal Education[MeSH Major Topic]) OR (Prenatal Education[MeSH Subheading])) OR (Prenatal Education[MeSH Terms]))) OR (((Education, Nonprofessional[MeSH Major Topic]) OR (Education, Nonprofessional[MeSH Subheading])) OR (Education, Nonprofessional[MeSH Terms])))) AND ((((((((baby formula feed*[Title/Abstract]) OR (infant formula feed*[Title/Abstract])) OR (baby milk feed*[Title/Abstract])) OR (formula feed*[Title/Abstract])) OR (bottle feed*[Title/Abstract])) OR (formula feeding[Title/Abstract])) OR (((bottle feeding[MeSH Major Topic]) OR (bottle feeding[MeSH Subheading])) OR (bottle feeding[MeSH Terms]))) OR (((infant formula[MeSH Major Topic]) OR (infant formula[MeSH Subheading])) OR (infant formula[MeSH Terms])))	504
#33	Search: ((((((((((experiment[Title/Abstract]) OR (treatment[Title/Abstract])) OR (intervention[Title/Abstract])) OR (((Clinical Trial[MeSH Major Topic]) OR (Clinical Trial[MeSH Subheading])) OR (Clinical Trial[MeSH Terms]))) OR (study[Title/Abstract])) OR (Program[Title/Abstract])) OR	11,382,802

Search	Query	Results
	(((Program[MeSH Major Topic]) OR (Program[MeSH Subheading])) OR (Program[MeSH Terms]))) OR (trial[Title/Abstract])) OR (((Pragmatic Clinical Trials as Topic[MeSH Major Topic]) OR (Pragmatic Clinical Trials as Topic[MeSH Subheading])) OR (Pragmatic Clinical Trials as Topic[MeSH Terms]))) OR (((Non-Randomized Controlled Trials as Topic[MeSH Major Topic]) OR (Non-Randomized Controlled Trials as Topic[MeSH Subheading])) OR (Non-Randomized Controlled Trials as Topic[MeSH Terms]))) OR (((Clinical Trials as Topic[MeSH Major Topic]) OR (Clinical Trials as Topic[MeSH Subheading])) OR (Clinical Trials as Topic[MeSH Terms]))) OR (((Randomized Controlled Trials as Topic[MeSH Major Topic]) OR (Randomized Controlled Trials as Topic[MeSH Subheading])) OR (Randomized Controlled Trials as Topic[MeSH Terms]))	
#32	Search: experiment[Title/Abstract]	321,264
#31	Search: treatment[Title/Abstract]	4,321,933
#30	Search: intervention[Title/Abstract]	608,617
#29	Search: ((Clinical Trial[MeSH Major Topic]) OR (Clinical Trial[MeSH Subheading])) OR (Clinical Trial[MeSH Terms])	344,332
#28	Search: study[Title/Abstract]	7,833,021
#27	Search: Program[Title/Abstract]	448,864
#26	Search: ((Program[MeSH Major Topic]) OR (Program[MeSH Subheading])) OR (Program[MeSH Terms])	0
#25	Search: trial[Title/Abstract]	606,825
#24	Search: ((Pragmatic Clinical Trials as Topic[MeSH Major Topic]) OR (Pragmatic Clinical Trials as Topic[MeSH Subheading])) OR (Pragmatic Clinical Trials as Topic[MeSH Terms])	464
#23	Search: ((Non-Randomized Controlled Trials as Topic[MeSH Major Topic]) OR (Non-Randomized Controlled Trials as Topic[MeSH Subheading])) OR (Non-Randomized Controlled Trials as Topic[MeSH Terms])	733
#22	Search: ((Clinical Trials as Topic[MeSH Major Topic]) OR (Clinical Trials as Topic[MeSH Subheading])) OR (Clinical Trials as Topic[MeSH Terms])	344,332

Search	Query	Results
#21	Search: ((Randomized Controlled Trials as Topic[MeSH Major Topic]) OR (Randomized Controlled Trials as Topic[MeSH Subheading])) OR (Randomized Controlled Trials as Topic[MeSH Terms])	138,364
#20	Search: (((((((((Support Groups[MeSH Major Topic]) OR (Support Groups[MeSH Subheading])) OR (Support Groups[MeSH Terms])) OR (((Social Support[MeSH Major Topic]) OR (Social Support[MeSH Subheading])) OR (Social Support[MeSH Terms]))) OR (parent* support*[Title/Abstract])) OR (parent* class[Title/Abstract])) OR (((Patient Education Handout[MeSH Major Topic]) OR (Patient Education Handout[MeSH Subheading])) OR (Patient Education Handout[MeSH Terms]))) OR (((Patient Education as Topic[MeSH Major Topic]) OR (Patient Education as Topic[MeSH Subheading])) OR (Patient Education as Topic[MeSH Terms]))) OR (((Health Education[MeSH Major Topic]) OR (Health Education[MeSH Subheading])) OR (Health Education[MeSH Terms]))) OR (((Prenatal Education[MeSH Major Topic]) OR (Prenatal Education[MeSH Subheading])) OR (Prenatal Education[MeSH Terms]))) OR (((Education, Nonprofessional[MeSH Major Topic]) OR (Education, Nonprofessional[MeSH Subheading])) OR (Education, Nonprofessional[MeSH Terms]))	467,907
#19	Search: ((Support Groups[MeSH Major Topic]) OR (Support Groups[MeSH Subheading])) OR (Support Groups[MeSH Terms])	10,194
#18	Search: ((Social Support[MeSH Major Topic]) OR (Social Support[MeSH Subheading])) OR (Social Support[MeSH Terms])	71,216
#17	Search: parent* support*[Title/Abstract]	535
#16	Search: parent* class[Title/Abstract]	10,543
#15	Search: ((Patient Education Handout[MeSH Major Topic]) OR (Patient Education Handout[MeSH Subheading])) OR (Patient Education Handout[MeSH Terms])	0
#14	Search: ((Patient Education as Topic[MeSH Major Topic]) OR (Patient Education as Topic[MeSH Subheading])) OR (Patient Education as Topic[MeSH Terms])	85,474
#13	Search: ((Health Education[MeSH Major Topic]) OR (Health Education[MeSH Subheading])) OR (Health Education[MeSH Terms])	360,500
#12	Search: ((Prenatal Education[MeSH Major Topic]) OR (Prenatal Education[MeSH Subheading])) OR (Prenatal Education[MeSH Terms])	2,340

Search	Query	Results
#11	Search: ((Education, Nonprofessional[MeSH Major Topic]) OR (Education, Nonprofessional[MeSH Subheading])) OR (Education, Nonprofessional[MeSH Terms])	273,386
#10	Search: (((((((baby formula feed*[Title/Abstract]) OR (infant formula feed*[Title/Abstract])) OR (baby milk feed*[Title/Abstract])) OR (formula feed*[Title/Abstract])) OR (bottle feed*[Title/Abstract])) OR (formula feeding[Title/Abstract])) OR (((bottle feeding[MeSH Major Topic]) OR (bottle feeding[MeSH Subheading])) OR (bottle feeding[MeSH Terms]))) OR (((infant formula[MeSH Major Topic]) OR (infant formula[MeSH Subheading])) OR (infant formula[MeSH Terms]))	11,167
#9	Search: baby formula feed*[Title/Abstract]	1,588
#8	Search: infant formula feed*[Title/Abstract]	91
#7	Search: baby milk feed*[Title/Abstract]	1,010
#6	Search: formula feed*[Title/Abstract]	1,907
#5	Search: bottle feed*[Title/Abstract]	1,925
#4	Search: formula feeding[Title/Abstract]	1,616
#2	Search: ((bottle feeding[MeSH Major Topic]) OR (bottle feeding[MeSH Subheading])) OR (bottle feeding[MeSH Terms])	3,810
#1	Search: ((infant formula[MeSH Major Topic]) OR (infant formula[MeSH Subheading])) OR (infant formula[MeSH Terms])	4,556

PubMed, 10/8/20, search terms and key words for ‘infant formula’ + search terms and key words for ‘parent education’ + search terms and key words on programs, studies and interventions

Search	Query	Results
#7	Search: ((((((((baby formula feed*[Title/Abstract]) OR (infant formula feed*[Title/Abstract])) OR (baby milk feed*[Title/Abstract])) OR (formula feed*[Title/Abstract])) OR (bottle feed*[Title/Abstract])) OR (formula feeding[Title/Abstract])) OR (((bottle feeding[MeSH Major Topic]) OR (bottle feeding[MeSH Subheading])) OR (bottle feeding[MeSH Terms]))) OR (((infant formula[MeSH Major Topic]) OR (infant formula[MeSH Subheading])) OR (infant formula[MeSH Terms]))) AND (((((((((((experiment[Title/Abstract]) OR (treatment[Title/Abstract])) OR (intervention[Title/Abstract])) OR (((Clinical Trial[MeSH Major Topic]) OR (Clinical Trial[MeSH Subheading])) OR (Clinical Trial[MeSH Terms]))) OR (study[Title/Abstract])) OR (Program[Title/Abstract])) OR (((Program[MeSH Major Topic]) OR (Program[MeSH Subheading])) OR (Program[MeSH Terms]))) OR (trial[Title/Abstract])) OR (((Pragmatic Clinical Trials as Topic[MeSH Major Topic]) OR (Pragmatic Clinical Trials as Topic[MeSH Subheading])) OR (Pragmatic Clinical Trials as Topic[MeSH Terms]))) OR (((Non-Randomized Controlled Trials as Topic[MeSH Major Topic]) OR (Non-Randomized Controlled Trials as Topic[MeSH Subheading])) OR (Non-Randomized Controlled Trials as Topic[MeSH Terms]))) OR (((Clinical Trials as Topic[MeSH Major Topic]) OR (Clinical Trials as Topic[MeSH Subheading])) OR (Clinical Trials as Topic[MeSH Terms]))) OR (((Randomized Controlled Trials as Topic[MeSH Major Topic]) OR (Randomized Controlled Trials as Topic[MeSH Subheading])) OR (Randomized Controlled Trials as Topic[MeSH Terms])))) AND (((tooth decay[Title/Abstract]) OR ((((Dental Care[MeSH Major Topic]) OR (Dental Care[MeSH Subheading])) OR (Dental Care[MeSH Terms])) OR (Dental Care[Title/Abstract]))) OR ((((Dental Caries[MeSH Major Topic]) OR (Dental Caries[MeSH Subheading])) OR (Dental Caries[MeSH Terms])) OR (Dental Caries[Title/Abstract])))	306
#6	Search: ((tooth decay[Title/Abstract]) OR ((((Dental Care[MeSH Major Topic]) OR (Dental Care[MeSH Subheading])) OR (Dental Care[MeSH Terms])) OR (Dental Care[Title/Abstract]))) OR ((((Dental Caries[MeSH Major Topic]) OR (Dental Caries[MeSH Subheading])) OR (Dental Caries[MeSH Terms])) OR (Dental Caries[Title/Abstract]))	85,887
#5	Search: tooth decay[Title/Abstract]	1,288
#4	Search: (((Dental Care[MeSH Major Topic]) OR (Dental Care[MeSH Subheading])) OR (Dental Care[MeSH Terms])) OR (Dental Care[Title/Abstract])	39,613

Search	Query	Results
#3	Search: (((Dental Caries[MeSH Major Topic]) OR (Dental Caries[MeSH Subheading])) OR (Dental Caries[MeSH Terms])) OR (Dental Caries[Title/Abstract])	51,406
#2	Search: (((((((((((experiment[Title/Abstract]) OR (treatment[Title/Abstract])) OR (intervention[Title/Abstract])) OR (((Clinical Trial[MeSH Major Topic]) OR (Clinical Trial[MeSH Subheading])) OR (Clinical Trial[MeSH Terms]))) OR (study[Title/Abstract])) OR (Program[Title/Abstract])) OR (((Program[MeSH Major Topic]) OR (Program[MeSH Subheading])) OR (Program[MeSH Terms]))) OR (trial[Title/Abstract])) OR (((Pragmatic Clinical Trials as Topic[MeSH Major Topic]) OR (Pragmatic Clinical Trials as Topic[MeSH Subheading])) OR (Pragmatic Clinical Trials as Topic[MeSH Terms]))) OR (((Non-Randomized Controlled Trials as Topic[MeSH Major Topic]) OR (Non-Randomized Controlled Trials as Topic[MeSH Subheading])) OR (Non-Randomized Controlled Trials as Topic[MeSH Terms]))) OR (((Clinical Trials as Topic[MeSH Major Topic]) OR (Clinical Trials as Topic[MeSH Subheading])) OR (Clinical Trials as Topic[MeSH Terms]))) OR (((Randomized Controlled Trials as Topic[MeSH Major Topic]) OR (Randomized Controlled Trials as Topic[MeSH Subheading])) OR (Randomized Controlled Trials as Topic[MeSH Terms]))	11,382,802
#1	Search: (((((((baby formula feed*[Title/Abstract]) OR (infant formula feed*[Title/Abstract])) OR (baby milk feed*[Title/Abstract])) OR (formula feed*[Title/Abstract])) OR (bottle feed*[Title/Abstract])) OR (formula feeding[Title/Abstract])) OR (((bottle feeding[MeSH Major Topic]) OR (bottle feeding[MeSH Subheading])) OR (bottle feeding[MeSH Terms]))) OR (((infant formula[MeSH Major Topic]) OR (infant formula[MeSH Subheading])) OR (infant formula[MeSH Terms]))	11,167

PubMed, 10/8/20, search terms and key words for ‘infant formula’ + search terms and key words for ‘dental caries’ + search terms and key words on programs, studies and interventions

Search	Query	Results
#5	Search: ((stop*[Text Word] OR quit*[Text Word] OR cessation[Text Word] OR cease[Text Word] OR wean[Text Word]) AND (bottle OR formula)) AND ((((((((baby formula feed*[Title/Abstract]) OR (infant formula feed*[Title/Abstract])) OR (baby milk feed*[Title/Abstract])) OR (formula feed*[Title/Abstract])) OR (bottle feed*[Title/Abstract])) OR (formula feeding[Title/Abstract])) OR (((bottle feeding[MeSH Major Topic]) OR (bottle feeding[MeSH Subheading])) OR (bottle feeding[MeSH Terms]))) OR (((infant formula[MeSH Major Topic]) OR (infant formula[MeSH Subheading])) OR (infant formula[MeSH Terms])))	374
#4	Search: (stop*[Text Word] OR quit*[Text Word] OR cessation[Text Word] OR cease[Text Word] OR wean[Text Word]) AND (bottle OR formula)	2,170
#3	Search: bottle OR formula	131,401
#2	Search: stop*[Text Word] OR quit*[Text Word] OR cessation[Text Word] OR cease[Text Word] OR wean[Text Word]	306,400
#1	Search: (((((((baby formula feed*[Title/Abstract]) OR (infant formula feed*[Title/Abstract])) OR (baby milk feed*[Title/Abstract])) OR (formula feed*[Title/Abstract])) OR (bottle feed*[Title/Abstract])) OR (formula feeding[Title/Abstract])) OR (((bottle feeding[MeSH Major Topic]) OR (bottle feeding[MeSH Subheading])) OR (bottle feeding[MeSH Terms]))) OR (((infant formula[MeSH Major Topic]) OR (infant formula[MeSH Subheading])) OR (infant formula[MeSH Terms]))	11,167

PubMed, 10/8/20, search terms and key words for ‘infant formula’ + key words for ‘stopping’

Set	Results	
# 22	1,665	#18 AND #17 AND #11<br><br>**Refined by:** [excluding] **WEB OF SCIENCE CATEGORIES:** (AGRICULTURE DAIRY ANIMAL SCIENCE OR ENGINEERING ENVIRONMENTAL OR OCEANOGRAPHY OR AGRICULTURE MULTIDISCIPLINARY OR ENGINEERING MULTIDISCIPLINARY OR ONCOLOGY OR ENTOMOLOGY OR OPHTHALMOLOGY OR ANESTHESIOLOGY OR ENVIRONMENTAL SCIENCES OR OTORHINOLARYNGOLOGY OR ENVIRONMENTAL STUDIES OR EVOLUTIONARY BIOLOGY OR BIOCHEMISTRY MOLECULAR BIOLOGY OR BIODIVERSITY CONSERVATION OR FISHERIES OR POLYMER SCIENCE OR BIOLOGY OR BIOTECHNOLOGY APPLIED MICROBIOLOGY OR GENETICS HEREDITY OR CARDIAC CARDIOVASCULAR SYSTEMS OR GERIATRICS GERONTOLOGY OR CHEMISTRY APPLIED OR GERONTOLOGY OR CHEMISTRY MULTIDISCIPLINARY OR GREEN SUSTAINABLE SCIENCE TECHNOLOGY OR CHEMISTRY PHYSICAL OR CLINICAL NEUROLOGY OR COMPUTER SCIENCE ARTIFICIAL INTELLIGENCE OR IMMUNOLOGY OR COMPUTER SCIENCE INFORMATION SYSTEMS OR INFECTIOUS DISEASES OR PUBLIC ENVIRONMENTAL OCCUPATIONAL HEALTH OR COMPUTER SCIENCE INTERDISCIPLINARY APPLICATIONS OR RADIOLOGY NUCLEAR MEDICINE MEDICAL IMAGING OR COMPUTER SCIENCE SOFTWARE ENGINEERING OR COMPUTER SCIENCE THEORY METHODS OR LINGUISTICS OR RESPIRATORY SYSTEM OR MANAGEMENT OR RHEUMATOLOGY OR MARINE FRESHWATER BIOLOGY OR DERMATOLOGY OR ECOLOGY OR ECONOMICS OR SUBSTANCE ABUSE OR MICROBIOLOGY OR SURGERY OR TOXICOLOGY OR NANOSCIENCE NANOTECHNOLOGY OR TROPICAL MEDICINE OR NEUROSCIENCES OR UROLOGY NEPHROLOGY OR VETERINARY SCIENCES OR ENERGY FUELS OR WATER RESOURCES OR ENGINEERING CHEMICAL OR ZOOLOGY OR ENGINEERING ELECTRICAL ELECTRONIC) AND [excluding] **WEB OF SCIENCE CATEGORIES:** (ACOUSTICS OR ENGINEERING MECHANICAL OR OPERATIONS RESEARCH MANAGEMENT SCIENCE OR AGRONOMY OR ENGINEERING PETROLEUM OR ORNITHOLOGY OR ERGONOMICS OR ORTHOPEDICS OR ANATOMY MORPHOLOGY OR ARCHITECTURE OR AREA STUDIES OR FORESTRY OR PLANT SCIENCES OR POLITICAL SCIENCE OR ASTRONOMY ASTROPHYSICS OR GEOCHEMISTRY GEOPHYSICS OR GEOGRAPHY OR GEOLOGY OR GEOSCIENCES MULTIDISCIPLINARY OR BIOCHEMICAL RESEARCH METHODS OR BIOPHYSICS OR BUSINESS OR HISTORY OR CHEMISTRY ANALYTICAL OR HISTORY PHILOSOPHY OF SCIENCE OR CHEMISTRY INORGANIC NUCLEAR OR HOSPITALITY LEISURE SPORT TOURISM OR CHEMISTRY MEDICINAL OR INDUSTRIAL RELATIONS LABOR OR COMPUTER SCIENCE CYBERNETICS OR PUBLIC ADMINISTRATION OR CONSTRUCTION BUILDING TECHNOLOGY OR INSTRUMENTS INSTRUMENTATION OR CRIMINOLOGY PENOLOGY OR LAW OR ROBOTICS OR MATERIALS SCIENCE CERAMICS OR MATERIALS SCIENCE CHARACTERIZATION TESTING OR EDUCATION EDUCATIONAL RESEARCH OR MATHEMATICAL COMPUTATIONAL BIOLOGY OR EDUCATION SCIENTIFIC DISCIPLINES OR EDUCATION SPECIAL OR SOIL SCIENCE OR SPORT SCIENCES OR ENGINEERING BIOMEDICAL OR TRANSPLANTATION OR ENGINEERING CIVIL OR TRANSPORTATION OR ENGINEERING INDUSTRIAL OR TRANSPORTATION SCIENCE TECHNOLOGY OR ENGINEERING MANUFACTURING) AND [excluding] **WEB OF SCIENCE CATEGORIES:** (MEDICINE LEGAL OR AUTOMATION CONTROL SYSTEMS OR OPTICS OR RELIGION OR PARASITOLOGY OR REMOTE SENSING OR PERIPHERAL VASCULAR DISEASE OR SPECTROSCOPY OR URBAN STUDIES OR VIROLOGY)<br><br>*Indexes=SCI-EXPANDED, SSCI, A&HCI, CPCI-S, CPCI-SSH, ESCI, CCR-EXPANDED, IC Timespan=All years*
# 21	1,675	#18 AND #17 AND #11<br><br>**Refined by:** [excluding] **WEB OF SCIENCE CATEGORIES:** (AGRICULTURE DAIRY ANIMAL SCIENCE OR ENGINEERING ENVIRONMENTAL OR OCEANOGRAPHY OR AGRICULTURE MULTIDISCIPLINARY OR ENGINEERING MULTIDISCIPLINARY OR ONCOLOGY OR ENTOMOLOGY OR OPHTHALMOLOGY OR ANESTHESIOLOGY OR ENVIRONMENTAL SCIENCES OR OTORHINOLARYNGOLOGY OR ENVIRONMENTAL STUDIES OR EVOLUTIONARY BIOLOGY OR BIOCHEMISTRY MOLECULAR BIOLOGY OR BIODIVERSITY CONSERVATION OR FISHERIES OR POLYMER SCIENCE OR BIOLOGY OR BIOTECHNOLOGY APPLIED MICROBIOLOGY OR GENETICS HEREDITY OR CARDIAC CARDIOVASCULAR SYSTEMS OR GERIATRICS GERONTOLOGY OR CHEMISTRY APPLIED OR GERONTOLOGY OR CHEMISTRY MULTIDISCIPLINARY OR GREEN SUSTAINABLE SCIENCE TECHNOLOGY OR CHEMISTRY PHYSICAL OR CLINICAL NEUROLOGY OR COMPUTER SCIENCE

ARTIFICIAL INTELLIGENCE OR IMMUNOLOGY OR COMPUTER SCIENCE INFORMATION SYSTEMS OR INFECTIOUS DISEASES OR PUBLIC ENVIRONMENTAL OCCUPATIONAL HEALTH OR COMPUTER SCIENCE INTERDISCIPLINARY APPLICATIONS OR RADIOLOGY NUCLEAR MEDICINE MEDICAL IMAGING OR COMPUTER SCIENCE SOFTWARE ENGINEERING OR COMPUTER SCIENCE THEORY METHODS OR LINGUISTICS OR RESPIRATORY SYSTEM OR MANAGEMENT OR RHEUMATOLOGY OR MARINE FRESHWATER BIOLOGY OR DERMATOLOGY OR ECOLOGY OR ECONOMICS OR SUBSTANCE ABUSE OR MICROBIOLOGY OR SURGERY OR TOXICOLOGY OR NANOSCIENCE NANOTECHNOLOGY OR TROPICAL MEDICINE OR NEUROSCIENCES OR UROLOGY NEPHROLOGY OR VETERINARY SCIENCES OR ENERGY FUELS OR WATER RESOURCES OR ENGINEERING CHEMICAL OR ZOOLOGY OR ENGINEERING ELECTRICAL ELECTRONIC) AND [excluding] **WEB OF SCIENCE CATEGORIES:** (ACOUSTICS OR ENGINEERING MECHANICAL OR OPERATIONS RESEARCH MANAGEMENT SCIENCE OR AGRONOMY OR ENGINEERING PETROLEUM OR ORNITHOLOGY OR ERGONOMICS OR ORTHOPEDICS OR ANATOMY MORPHOLOGY OR ARCHITECTURE OR AREA STUDIES OR FORESTRY OR PLANT SCIENCES OR POLITICAL SCIENCE OR ASTRONOMY ASTROPHYSICS OR GEOCHEMISTRY GEOPHYSICS OR GEOGRAPHY OR GEOLOGY OR GEOSCIENCES MULTIDISCIPLINARY OR BIOCHEMICAL RESEARCH METHODS OR BIOPHYSICS OR BUSINESS OR HISTORY OR CHEMISTRY ANALYTICAL OR HISTORY PHILOSOPHY OF SCIENCE OR CHEMISTRY INORGANIC NUCLEAR OR HOSPITALITY LEISURE SPORT TOURISM OR CHEMISTRY MEDICINAL OR INDUSTRIAL RELATIONS LABOR OR COMPUTER SCIENCE CYBERNETICS OR PUBLIC ADMINISTRATION OR CONSTRUCTION BUILDING TECHNOLOGY OR INSTRUMENTS INSTRUMENTATION OR CRIMINOLOGY PENOLOGY OR LAW OR ROBOTICS OR MATERIALS SCIENCE CERAMICS OR MATERIALS SCIENCE CHARACTERIZATION TESTING OR EDUCATION EDUCATIONAL RESEARCH OR MATHEMATICAL COMPUTATIONAL BIOLOGY OR EDUCATION SCIENTIFIC DISCIPLINES OR EDUCATION SPECIAL OR SOIL SCIENCE OR SPORT SCIENCES OR ENGINEERING BIOMEDICAL OR TRANSPLANTATION OR ENGINEERING CIVIL OR TRANSPORTATION OR ENGINEERING INDUSTRIAL OR TRANSPORTATION SCIENCE TECHNOLOGY OR ENGINEERING MANUFACTURING)

Indexes=SCI-EXPANDED, SSCI, A&HCI, CPCI-S, CPCI-SSH, ESCI, CCR-EXPANDED, IC Timespan=All years

20 | 1,964 | #18 AND #17 AND #11

Refined by: [excluding] **WEB OF SCIENCE CATEGORIES:** (AGRICULTURE DAIRY ANIMAL SCIENCE OR ENGINEERING ENVIRONMENTAL OR OCEANOGRAPHY OR AGRICULTURE MULTIDISCIPLINARY OR ENGINEERING MULTIDISCIPLINARY OR ONCOLOGY OR ENTOMOLOGY OR OPHTHALMOLOGY OR ANESTHESIOLOGY OR ENVIRONMENTAL SCIENCES OR OTORHINOLARYNGOLOGY OR ENVIRONMENTAL STUDIES OR EVOLUTIONARY BIOLOGY OR BIOCHEMISTRY MOLECULAR BIOLOGY OR BIODIVERSITY CONSERVATION OR FISHERIES OR POLYMER SCIENCE OR BIOLOGY OR BIOTECHNOLOGY APPLIED MICROBIOLOGY OR GENETICS HEREDITY OR CARDIAC CARDIOVASCULAR SYSTEMS OR GERIATRICS GERONTOLOGY OR CHEMISTRY APPLIED OR GERONTOLOGY OR CHEMISTRY MULTIDISCIPLINARY OR GREEN SUSTAINABLE SCIENCE TECHNOLOGY OR CHEMISTRY PHYSICAL OR CLINICAL NEUROLOGY OR COMPUTER SCIENCE ARTIFICIAL INTELLIGENCE OR IMMUNOLOGY OR COMPUTER SCIENCE INFORMATION SYSTEMS OR INFECTIOUS DISEASES OR PUBLIC ENVIRONMENTAL OCCUPATIONAL HEALTH OR COMPUTER SCIENCE INTERDISCIPLINARY APPLICATIONS OR RADIOLOGY NUCLEAR MEDICINE MEDICAL IMAGING OR COMPUTER SCIENCE SOFTWARE ENGINEERING OR COMPUTER SCIENCE THEORY METHODS OR LINGUISTICS OR RESPIRATORY SYSTEM OR MANAGEMENT OR RHEUMATOLOGY OR MARINE FRESHWATER BIOLOGY OR DERMATOLOGY OR ECOLOGY OR ECONOMICS OR SUBSTANCE ABUSE OR MICROBIOLOGY OR SURGERY OR TOXICOLOGY OR NANOSCIENCE NANOTECHNOLOGY OR TROPICAL MEDICINE OR NEUROSCIENCES OR UROLOGY NEPHROLOGY OR VETERINARY SCIENCES OR ENERGY FUELS OR WATER RESOURCES OR ENGINEERING CHEMICAL OR ZOOLOGY OR ENGINEERING ELECTRICAL ELECTRONIC)

Indexes=SCI-EXPANDED, SSCI, A&HCI, CPCI-S, CPCI-SSH, ESCI, CCR-EXPANDED, IC Timespan=All years

19 | 3,143 | #18 AND #17 AND #11

Indexes=SCI-EXPANDED, SSCI, A&HCI, CPCI-S, CPCI-SSH, ESCI, CCR-EXPANDED, IC Timespan=All years

# 18	26,597,737	**TOPIC:** (program) *OR* **TOPIC:** (intervention) *OR* **TOPIC:** (training) *OR* **TOPIC:** (treatment) *OR* **TOPIC:** (education) *OR* **TOPIC:** (trial) *OR* **TOPIC:** (study) *OR* **TOPIC:** (RCT) *OR* **TOPIC:** (experiment) *OR* **TOPIC:** (research) *OR* **TOPIC:** (clinical trial)<br>*Indexes=SCI-EXPANDED, SSCI, A&HCI, CPCI-S, CPCI-SSH, ESCI, CCR-EXPANDED, IC Timespan=All years*
# 17	814,130	#16 OR #15 OR #14 OR #13 OR #12<br>*Indexes=SCI-EXPANDED, SSCI, A&HCI, CPCI-S, CPCI-SSH, ESCI, CCR-EXPANDED, IC Timespan=All years*
# 16	2,165	**TOPIC:** (prenatal class) *OR* **TOPIC:** (antenatal class)<br>*Indexes=SCI-EXPANDED, SSCI, A&HCI, CPCI-S, CPCI-SSH, ESCI, CCR-EXPANDED, IC Timespan=All years*
# 15	434,705	**TOPIC:** (support group)<br>*Indexes=SCI-EXPANDED, SSCI, A&HCI, CPCI-S, CPCI-SSH, ESCI, CCR-EXPANDED, IC Timespan=All years*
# 14	267,614	**TOPIC:** (social support)<br>*Indexes=SCI-EXPANDED, SSCI, A&HCI, CPCI-S, CPCI-SSH, ESCI, CCR-EXPANDED, IC Timespan=All years*
# 13	51,051	**TOPIC:** (parent* education)<br>*Indexes=SCI-EXPANDED, SSCI, A&HCI, CPCI-S, CPCI-SSH, ESCI, CCR-EXPANDED, IC Timespan=All years*
# 12	145,393	**TOPIC:** (patient education)<br>*Indexes=SCI-EXPANDED, SSCI, A&HCI, CPCI-S, CPCI-SSH, ESCI, CCR-EXPANDED, IC Timespan=All years*
# 11	127,054	#10 OR #9 OR #8 OR #7 OR #6 OR #5 OR #4 OR #3 OR #2 OR #1<br>*Indexes=SCI-EXPANDED, SSCI, A&HCI, CPCI-S, CPCI-SSH, ESCI, CCR-EXPANDED, IC Timespan=All years*
# 10	9,110	**TOPIC:** (milk powder or powder* milk)<br>*Indexes=SCI-EXPANDED, SSCI, A&HCI, CPCI-S, CPCI-SSH, ESCI, CCR-EXPANDED, IC Timespan=All years*
# 9	75,845	**TOPIC:** (mix* feed*)<br>*Indexes=SCI-EXPANDED, SSCI, A&HCI, CPCI-S, CPCI-SSH, ESCI, CCR-EXPANDED, IC Timespan=All years*
# 8	25,296	**TOPIC:** (infant formula or formula feed* or formula milk)<br>*Indexes=SCI-EXPANDED, SSCI, A&HCI, CPCI-S, CPCI-SSH, ESCI, CCR-EXPANDED, IC Timespan=All years*
# 7	1,403	**TOPIC:** (baby milk feed*)<br>*Indexes=SCI-EXPANDED, SSCI, A&HCI, CPCI-S, CPCI-SSH, ESCI, CCR-EXPANDED, IC Timespan=All years*
# 6	16,180	**TOPIC:** (bottle use*)<br>*Indexes=SCI-EXPANDED, SSCI, A&HCI, CPCI-S, CPCI-SSH, ESCI, CCR-EXPANDED, IC Timespan=All years*
# 5	12,404	**TOPIC:** (formula feed*)<br>*Indexes=SCI-EXPANDED, SSCI, A&HCI, CPCI-S, CPCI-SSH, ESCI, CCR-EXPANDED, IC Timespan=All years*

# 4	3,429	**TOPIC:** (bottle feed*)<br>*Indexes=SCI-EXPANDED, SSCI, A&HCI, CPCI-S, CPCI-SSH, ESCI, CCR-EXPANDED, IC Timespan=All years*
# 3	981	**TOPIC:** (baby bottle)<br>*Indexes=SCI-EXPANDED, SSCI, A&HCI, CPCI-S, CPCI-SSH, ESCI, CCR-EXPANDED, IC Timespan=All years*
# 2	13,214	**TOPIC:** (formula feeding)<br>*Indexes=SCI-EXPANDED, SSCI, A&HCI, CPCI-S, CPCI-SSH, ESCI, CCR-EXPANDED, IC Timespan=All years*
# 1	16,981	**TOPIC:** (infant formula)<br>*Indexes=SCI-EXPANDED, SSCI, A&HCI, CPCI-S, CPCI-SSH, ESCI, CCR-EXPANDED, IC Timespan=All years*

Web of Science, 3/2/21, key words for ‘infant formula’ + key words for ‘parent education’ + search terms and key words on programs, studies and interventions + exclusion of non-relevant academic fields

# 13	276	#12 AND #11<br>*Indexes=SCI-EXPANDED, SSCI, A&HCI, CPCI-S, CPCI-SSH, ESCI, CCR-EXPANDED, IC Timespan=All years*
# 12	866	**TOPIC:** ((stop* OR quit* OR cessation OR cease OR wean) NEAR/3 (bottle* OR formula))<br>*Indexes=SCI-EXPANDED, SSCI, A&HCI, CPCI-S, CPCI-SSH, ESCI, CCR-EXPANDED, IC Timespan=All years*
# 11	127,081	#10 OR #9 OR #8 OR #7 OR #6 OR #5 OR #4 OR #3 OR #2 OR #1<br>*Indexes=SCI-EXPANDED, SSCI, A&HCI, CPCI-S, CPCI-SSH, ESCI, CCR-EXPANDED, IC Timespan=All years*
# 10	9,112	**TOPIC:** (milk powder or powder* milk)<br>*Indexes=SCI-EXPANDED, SSCI, A&HCI, CPCI-S, CPCI-SSH, ESCI, CCR-EXPANDED, IC Timespan=All years*
# 9	75,865	**TOPIC:** (mix* feed*)<br>*Indexes=SCI-EXPANDED, SSCI, A&HCI, CPCI-S, CPCI-SSH, ESCI, CCR-EXPANDED, IC Timespan=All years*
# 8	25,297	**TOPIC:** (infant formula or formula feed* or formula milk)<br>*Indexes=SCI-EXPANDED, SSCI, A&HCI, CPCI-S, CPCI-SSH, ESCI, CCR-EXPANDED, IC Timespan=All years*
# 7	1,403	**TOPIC:** (baby milk feed*)<br>*Indexes=SCI-EXPANDED, SSCI, A&HCI, CPCI-S, CPCI-SSH, ESCI, CCR-EXPANDED, IC Timespan=All years*
# 6	16,184	**TOPIC:** (bottle use*)<br>*Indexes=SCI-EXPANDED, SSCI, A&HCI, CPCI-S, CPCI-SSH, ESCI, CCR-EXPANDED, IC Timespan=All years*
# 5	12,404	**TOPIC:** (formula feed*)<br>*Indexes=SCI-EXPANDED, SSCI, A&HCI, CPCI-S, CPCI-SSH, ESCI, CCR-EXPANDED, IC Timespan=All years*
# 4	3,429	**TOPIC:** (bottle feed*)<br>*Indexes=SCI-EXPANDED, SSCI, A&HCI, CPCI-S, CPCI-SSH, ESCI, CCR-EXPANDED, IC Timespan=All years*
# 3	981	**TOPIC:** (baby bottle)<br>*Indexes=SCI-EXPANDED, SSCI, A&HCI, CPCI-S, CPCI-SSH, ESCI, CCR-EXPANDED, IC Timespan=All years*
# 2	13,214	**TOPIC:** (formula feeding)

		Indexes=SCI-EXPANDED, SSCI, A&HCI, CPCI-S, CPCI-SSH, ESCI, CCR-EXPANDED, IC Timespan=All years
# 1	16,982	**TOPIC:** (infant formula) *Indexes=SCI-EXPANDED, SSCI, A&HCI, CPCI-S, CPCI-SSH, ESCI, CCR-EXPANDED, IC Timespan=All years*

Web of Science, 4/2/21, key words for ‘infant formula’ + key words for ‘stopping’

# 17	405	#16 AND #15 AND #11<br>*Indexes=SCI-EXPANDED, SSCI, A&HCI, CPCI-S, CPCI-SSH, ESCI, CCR-EXPANDED, IC Timespan=All years*
# 16	26,602,857	**TOPIC:** (program) *OR* **TOPIC:** (intervention) *OR* **TOPIC:** (training) *OR* **TOPIC:** (treatment) *OR* **TOPIC:** (education) *OR* **TOPIC:** (trial) *OR* **TOPIC:** (study) *OR* **TOPIC:** (RCT) *OR* **TOPIC:** (experiment) *OR* **TOPIC:** (research) *OR* **TOPIC:** (clinical trial)<br>*Indexes=SCI-EXPANDED, SSCI, A&HCI, CPCI-S, CPCI-SSH, ESCI, CCR-EXPANDED, IC Timespan=All years*
# 15	46,111	#14 OR #13 OR #12<br>*Indexes=SCI-EXPANDED, SSCI, A&HCI, CPCI-S, CPCI-SSH, ESCI, CCR-EXPANDED, IC Timespan=All years*
# 14	5,017	**TOPIC:** (tooth decay)<br>*Indexes=SCI-EXPANDED, SSCI, A&HCI, CPCI-S, CPCI-SSH, ESCI, CCR-EXPANDED, IC Timespan=All years*
# 13	21,387	**TOPIC:** (dental care)<br>*Indexes=SCI-EXPANDED, SSCI, A&HCI, CPCI-S, CPCI-SSH, ESCI, CCR-EXPANDED, IC Timespan=All years*
# 12	27,589	**TOPIC:** (dental caries)<br>*Indexes=SCI-EXPANDED, SSCI, A&HCI, CPCI-S, CPCI-SSH, ESCI, CCR-EXPANDED, IC Timespan=All years*
# 11	127,081	#10 OR #9 OR #8 OR #7 OR #6 OR #5 OR #4 OR #3 OR #2 OR #1<br>*Indexes=SCI-EXPANDED, SSCI, A&HCI, CPCI-S, CPCI-SSH, ESCI, CCR-EXPANDED, IC Timespan=All years*
# 10	9,112	**TOPIC:** (milk powder or powder* milk)<br>*Indexes=SCI-EXPANDED, SSCI, A&HCI, CPCI-S, CPCI-SSH, ESCI, CCR-EXPANDED, IC Timespan=All years*
# 9	75,865	**TOPIC:** (mix* feed*)<br>*Indexes=SCI-EXPANDED, SSCI, A&HCI, CPCI-S, CPCI-SSH, ESCI, CCR-EXPANDED, IC Timespan=All years*
# 8	25,297	**TOPIC:** (infant formula or formula feed* or formula milk)<br>*Indexes=SCI-EXPANDED, SSCI, A&HCI, CPCI-S, CPCI-SSH, ESCI, CCR-EXPANDED, IC Timespan=All years*
# 7	1,403	**TOPIC:** (baby milk feed*)<br>*Indexes=SCI-EXPANDED, SSCI, A&HCI, CPCI-S, CPCI-SSH, ESCI, CCR-EXPANDED, IC Timespan=All years*
# 6	16,184	**TOPIC:** (bottle use*)

Indexes=SCI-EXPANDED, SSCI, A&HCI, CPCI-S, CPCI-SSH, ESCI, CCR-EXPANDED, IC Timespan=All years

# 5	12,404	**TOPIC:** (formula feed*)<br>*Indexes=SCI-EXPANDED, SSCI, A&HCI, CPCI-S, CPCI-SSH, ESCI, CCR-EXPANDED, IC Timespan=All years*
# 4	3,429	**TOPIC:** (bottle feed*)<br>*Indexes=SCI-EXPANDED, SSCI, A&HCI, CPCI-S, CPCI-SSH, ESCI, CCR-EXPANDED, IC Timespan=All years*
# 3	981	**TOPIC:** (baby bottle)<br>*Indexes=SCI-EXPANDED, SSCI, A&HCI, CPCI-S, CPCI-SSH, ESCI, CCR-EXPANDED, IC Timespan=All years*
# 2	13,214	**TOPIC:** (formula feeding)<br>*Indexes=SCI-EXPANDED, SSCI, A&HCI, CPCI-S, CPCI-SSH, ESCI, CCR-EXPANDED, IC Timespan=All years*
# 1	16,982	**TOPIC:** (infant formula)<br>*Indexes=SCI-EXPANDED, SSCI, A&HCI, CPCI-S, CPCI-SSH, ESCI, CCR-EXPANDED, IC Timespan=All years*
