# Supplementary material for: Interventions Targeting Bottle and Formula Feeding in the Prevention and Treatment of Early Childhood Caries, Overweight and Obesity: An Integrative Review
Source: Int J Environ Res Public Health. 2021 Nov 23;18(23):12304. doi: 10.3390/ijerph182312304 (PMC8656950; doi:10.3390/ijerph182312304)
Supplement: Supplementary file 1 [file ijerph-18-12304-s001.zip › Suppt File 5. Excluded studies.pdf]

Supplementary Table 2: excluded studies that addressed formula feeding, bottle cessation or cup use briefly in the wider context of the study intervention.

Reference; study; country	Aim	Formula feeding or bottle cessation content	Reason for exclusion
Batliner et al. 2018 NCT01116726.<br><br>USA.	To test the efficacy of motivational interviewing (to improve the oral health of children in an American Indian population with increased risk of dental caries.	4 motivational interviewing sessions with a primary caregiver of an American Indian infant aged <3 mo. 8 motivational interviewing topics discussed: dental visits, water in sippy cups at bedtime, bottle cessation and cup transition at 1 yr, non-sugary food intake, oral bacteria causing caries, and oral hygiene.	Messages on bottle/cup use and avoidance of cariogenic foods are minimal (3 of 8 messages) in overall context of oral health.
Benitez et al. 1994<br><br>USA.	To determine whether a preventive approach focused on bottle use and daily fluoride treatment could stop ECC progression.	Instructions for teeth brushing, application of fluoride gel, bottle cessation and use of water in baby bottles, provided to caregivers of children aged 21-36 mo with dental caries lesions and history of bottle use.	Study was terminated after 3 mo, as ECC lesions increased in number and severity, with caregivers not complying with study protocol: "thus it was felt that the risks of continuing the study were high and the benefits to the children would be low."
Benjamin Neelon et al. 2014<br>*NAP SACC.*<br><br>USA.	To evaluate a pilot intervention to improve the nutrition and physical activity environments of child care centres for infants and toddlers.	A 6-month intervention was delivered to 32 licensed child care centres, to: assess the centre's nutrition and physical activity environments; select areas for improvement; and make changes. Of the 47 specific behavioural targets, two were "Solid foods not added to bottle" and "Bottles not propped/satiety cues respected".	Intervention was individualised to each child care centre's environment. Implementation strategies discussed by the interventionist was not guaranteed to address formula feeding, bottle use, or bottle cessation.
Blue et al. 2020.<br>*NCT04286256.*<br><br>USA.	To investigate if a motivational interviewing intervention improved dental caries risk-related behaviours of American Indian and Alaska Native caregivers of infants.	Over 4 well-child visits, American Indian and Alaska Native caregivers of ≤12 mo infants participated in motivational interviewing discussion of behaviour changes and desired oral health outcomes. Motivational interviewing topics were based on the caregivers' caries risk test, parental care of children's teeth QNR, and questions about caries risk and snacking habits.	Motivational interviewing intervention individualised to ECC risk-related behaviours. Oral health strategies discussed by the interventionist was not guaranteed to address formula feeding, bottle use, or bottle cessation.

Reference; study; country	Aim	Formula feeding or bottle cessation content	Reason for exclusion
Borrelli et al. 2019.<br><br>USA.	To test the effects of oral health text messages in improving paediatric oral health and parental attitudes.	Text messages with one core topic text message (tooth brushing, cleaning gums, visiting the dentist) and one choice topic text message (bedtime routine, bottle and sippy cup use, cariogenic beverage intake, healthy eating, using fluoride, fun facts) were sent to parents of children aged 6mo-7 yrs, over an 8 wk intervention period.	Text message topics about bottle and sippy cup use, cariogenic beverage intake and healthy eating were opt-in and parent-selected, and therefore not guaranteed to be received. Primary outcomes were on tooth brushing and fluoride use; outcomes related to dietary behaviour were limited to parental survey response on beliefs.
Brown et al. 2005. *Una Boca Saludable.*<br><br>USA.	To evaluate the effect of an educational program on knowledge and oral health behaviours, for Latino immigrant parents of school-aged children.	Two lessons on paediatric oral health, conducted in Spanish by a school nurse and bilingual dental instructor. Topics on dietary oral health promotion, oral hygiene, tooth brushing, and dental caries risk from bottle use.	Minimal focus on bottle cessation for dental caries prevention in context of wider oral health education topics. Results did not specify which areas of parental knowledge of oral health behaviours improved.
Chomitz et al. 2019 *Baby Steps to Health.*<br><br>USA.	To describe the design, feasibility, and acceptability of a dental caries prevention pilot study, conducted in an academic dental clinic among a primarily Asian immigrant population.	A brief motivational interviewing-informed intervention assessing obesity and dental caries risk in children 6-36 mo, where parents select one goal from 15 behavioural modification goals to improve feeding practices. Goals include: bottle transition to cup use; healthier bedtime feeding routines; limiting exposure to cariogenic drinks; decreased snacking and healthier meals; and healthier feeding practices.	Goals selected by caregivers does not guarantee bottle cessation or cariogenic behaviours would be addressed. 34% and 20% of carers selected goals on transitioning to healthier bedtime feeding routines and open cup use respectively, but the outcomes do not focus on if the goal was achieved.
Denney-Wilson et al. 2015.<br>Laws et al. 2018.<br>*Growing healthy.*<br><br>Australia.	To determine the feasibility and effectiveness of an mHealth obesity prevention intervention in terms of reach, acceptability, and impact	Parents of infants aged 0-9 mo accessed a free smartphone app and website with information on infant feeding, sleep and settling, and general support.<br><br>App messages included promotion of breastfeeding; promotion of best practice formula feeding, if not breastfeeding; delaying introduction of solids to ~6 mo	Minimal focus on best-practice formula feeding in context of best-practice infant feeding activities.

Reference; study; country	Aim	Formula feeding or bottle cessation content	Reason for exclusion
	on key infant feeding outcomes.	age; promotion of healthy first foods and infant feeding practices; and optimising fruit and vegetable exposure.	
Feldens et al. 2010. NCT00629629.<br><br>Brazil.	To investigate the effectiveness of home visits advising on healthy feeding, on the occurrence of dental caries at 4 years.	Home visiting to mothers giving birth in the public health system, from 10 days to 12 mo of infant age, on healthy eating advice, including not to use baby bottles as pacifiers and avoidance of added sugar and sugary foods in infant diet. Follow up for child's dental health at 4 yrs of age.	Minimal intervention focus is on bottle cessation and avoidance of cariogenic foods and beverages, in wider context of infant nutrition.
Freudenthal et al. 2010.<br><br>USA.	To examine if an individualized motivational interviewing approach to oral health education promoted positive changes caries-related behaviours.	Mothers received a motivational interviewing intervention, to facilitate dental caries risk-related behaviours for their 6-24mo child. Interventionist offered oral health strategies, based on mother's readiness for change. If mother asked, interventionists provided oral health information in the form of a menu. Telephone follow up made 1 and 2 wks later.	Motivational interviewing intervention individualised to ECC risk-related behaviours. Oral health strategies discussed by the interventionist was not guaranteed to address formula feeding, bottle use, or bottle cessation.
Harrison et al. 2010 [protocol] Harrison et al. 2011 [report] Harrison et al. 2012 *Kimaa Miywaapitet Nitawaashiim. ISRCTN41467632.*<br><br>Canada.	To test the effectiveness of motivational interviewing with mothers, to prevent ECC in Aboriginal Cree children.	Motivational interviewing session with expectant mothers between 12-34 wks pregnancy, or recently giving birth: one prenatal session and up to 6 postnatal sessions at well-child visits in community clinics. Mother could choose oral health behaviours to change from a menu of maternal and child oral health strategies. Child oral health strategies included: bottle cessation and cup use, safe fluids in cups, child soothing without food use, minimising juice intake, avoiding and limiting cariogenic snacks. Resource distribution: infant toothbrushes, toothpaste, sippy cups. Fluoride varnish application at 1 yr age.	Motivational interviewing intervention individualised to oral health strategies self-selected by the mother, and was not guaranteed to address bottle cessation or cariogenic behaviours.
King et al. 1998. *Healthy Smiles.*<br><br>USA.	To decrease the number of children who fell asleep with a bottle (with milk, formula or sugary drink)	Program description includes action planning and program implementation, with The University of Toledo and the Dental Center of Northwest Ohio.	No outcomes reported. Nil further about *Healthy Smiles* program in Ohio found.

Reference; study; country	Aim	Formula feeding or bottle cessation content	Reason for exclusion
	and increasing regular dental examination of children aged 2-5 yrs.	Action planning identified:<br>- children of adolescent mothers more vulnerable to BBTD → implemented parenting program for teens attending high school; distributed educational resources on preventing BBTD to elementary and high schools<br>- lower SES children with increased risk of BBTD → targeted education resources to day care centres, prenatal classes and paediatricians	
Machuca et al. 2016.<br>*Well Baby Group.*<br><br>USA.	To evaluate the effectiveness of a novel group well-child care intervention, as an alternative to traditional well-child care, for obesity prevention at age 2 years.	11 sessions of well-child care, from 1 to 18 mo of age, with group nutrition education together with 6-8 mother-child dyads, facilitated by paediatrician and dietitian. 30min nutrition education session included activities on responsive feeding practices – one activity is 'sippy cup use and spoon feeding'.	15 activities are included in 11 nutrition education sessions – one activity is 'sippy cup use and spoon feeding'. Messages on formula feeding, bottle use or bottle cessation are minimal, in overall context of infant feeding activities.
Matvieko-Sivar et al. 2019.<br>*CHErIsH.*<br><br>Ireland.	To examine acceptability and feasibility of the delivery of a brief infant feeding intervention by HCPs to parents at child vaccination visits.	A brief complex intervention, targeting parents to improve infant feeding behaviours, at 2-13 mo age, during vaccination visits. Verbal message delivery at each vaccination visit, on appropriate milk feeding and introduction to solid foods. At 2 mo and 4 mo visit, message on infant satiety is delivered.	Message on responsive feeding is brief and not specific to formula feeding: "Your baby gives you signals when they are hungry or full, like putting his/her hands to his/her mouth when hungry or turning away or falling asleep when full or not hungry. Crying does not always mean your baby is hungry".
Mattheus et al. 2020.<br><br>USA.	To assess the effect of oral health education on oral health beliefs and behaviours of attending WIC.	Initial visit with pregnant mother or family with child aged <5 yrs, with provision of dental supplies (toothbrush, toothpaste, dental floss) for family, and 5-7min oral health education. Education topics include: limiting sugar and juice, bottle cessation by 14 mo age; dental hygiene; fluoride varnish and toothpaste use; dental attendance every 6 months. Educational brochure distributed. Follow up visit at 3-6 months.	Messages on sugar avoidance and bottle cessation are minimal in overall context of oral health education.

Reference; study; country	Aim	Formula feeding or bottle cessation content	Reason for exclusion
Mohebbi et al. 2009.<br><br>Iran.	To evaluate the impact of a 6-month educational intervention on ECC.	Trial targeting mothers of 12-15 mo infants, at vaccinations at public health centres. Educational intervention for caries prevention in infants and toddlers, focusing on feeding habits, sugar intake, oral bacteria transmission and oral hygiene, using educational pamphlet, with (IG 1) without (IG 2) oral health instructions and telephone follow up reminders.	Messages on sugar avoidance and bottle cessation at night are minimal in overall context of ECC prevention.
Paul et al. 2014<br>Savage et al. 2016<br>Hohman et al. 2017<br>Savage et al. 2018<br>Ruggiero et al. 2020<br>*INSIGHT.*<br><br>USA.	To evaluate a responsive parenting intervention for responsive parenting and feeding, on obesity prevention.	Longitudinal randomised controlled trial, with delivery of intervention messages, from age 3-4 wks to 40 wks, and annual clinic visits at 1, 2 and 3 yrs.<br><br>Main messages to parents include: recognising infant hunger/satiety cues and feeding only in response to hunger; use non-feeding methods to soothe infants and toddlers; and allow children to determine the amount of food consumed. Includes limiting fruit juice intake; avoidance of sugar-sweetened drinks; how and when to transition bottle use to cup use; use of sippy cups without caloric drinks at non-meal times.	Messages on bottle use or bottle cessation is minimal in overall context of infant feeding, tummy time and active play.
Plutzer et al. 2008.<br><br>Australia.	To test the efficacy of an oral health promotion program for the parents of infants, starting during the pregnancy.	Anticipatory information education intervention, with three rounds of information: at 5-7 mo of pregnancy and infant 6 and 12 mo age. Round 1: oral hygiene during pregnancy, nutrition, pacifier use, infant sleeping patterns. Round 2 and 3: teeth eruption, oral hygiene, nutrition, healthy teeth development.	Unclear if intervention provided information on bottle use or bottle cessation.
Rai et al. 2019.<br><br>USA.	To examine effect of an oral health intervention to improve oral health knowledge, behaviour and self-efficacy of Mexican-	5 group intervention sessions with 7-10 caregivers and facilitator, with 15min skill-building exercise. Goal setting sheet with 9 goals, for caregivers to select two to achieve during 3-month intervention period. Goal options include: no infant sleeping with bottle; bottle cessation;	Goals selected by caregivers does not guarantee bottle cessation or cariogenic behaviours would be addressed. The least preferred behaviour change goals were bottle cessation and only using water in sippy cups, selected by 5 of 46 caregivers.

Reference; study; country	Aim	Formula feeding or bottle cessation content	Reason for exclusion
	American caregivers of children <6 years.	increase water intake, decrease juice and soda intake; only use water in sippy cups.	
Reifsnider et al. 2013<br>Reifsnider et al. 2018<br>*Preventing Childhood Obesity through Early Feeding and Parenting Guidance. NCT01905072.*<br><br>USA.	To examine the effectiveness of structured community health worker-provided home visits, to mothers in the WIC program, in preventing infant overweight and obesity in the first 3 years of life.	Community health workers delivers intervention through home visiting, from 36 wks pregnancy to infant 24 mo age. Intervention messages includes growth monitoring; exclusive breastfeeding, delay of solid feeding until 6 mo, appropriate portions for age, cease bottle use at 12 mo, limit bottle use to breast milk/infant formula/4oz of fruit juice, limit fruit juice intake, introducing cup use by 10-11 mo, avoiding sweetened drinks; parenting, activity and sleep.	Messages on bottle use or bottle cessation is minimal in overall context of infant feeding, play and parenting. No relevant outcomes on bottle use or bottle cessation reported.
Quiñonez et al., 2008.<br>*Into the Mouths of Babes.*<br><br>USA.	To describe the frequency and determinants of follow-up preventive oral health visits at medical offices among children screened for dental disease.	Longitudinal RCT, assessing the effectiveness of different types of continuing medical education on physician adoption of preventive dental services. Physicians in 2 IGs and 1 CG received continuing medical education, with additional support of fortnightly phone calls or in-office support with dental hygienist. Intervention: dental caries risk assessment, fluoride varnish, oral health education to caregivers.	Details of oral health education not specified.<br><br>2008 evaluation outcomes included use of bottle or sippy cup in bed as a risk factor for follow up dental care.
Watt et al. 2009.<br><br>United Kingdom.	To assess whether monthly home visits from trained volunteers could improve infant feeding practices at age 12 months.	Nine monthly sessions, from infant 3 to 12 mo age, delivered by trained volunteers on practical and non-judgemental support and advice to mothers through home visits. Topics focused on timing and introduction of solids and beverages, and cessation of bottle use, using UK government guidelines.	Messages on bottle cessation were minimal in the overall context of healthy infant feeding.
Weinstein et al. 2004.	To compare the effect of a motivational interviewing	Dental education targeted to Punjabi-speaking mothers of infants aged 6-18 mo.	Motivational interviewing intervention individualised to oral health strategies

Reference; study; country	Aim	Formula feeding or bottle cessation content	Reason for exclusion
Weinstein et al. 2006.<br><br>Canada.	counselling visit with traditional health education, for mothers of young children at high risk of developing dental caries.	CG received traditional ECC-prevention education: video and modified pamphlet, targeted to local South Asian Punjabi community. IG received CG education, with one motivational interviewing counselling session. Counselling involved mother's selection of caries-preventative behaviours to initiate from list of 7 topics, including: no adding sugar to bottles; cuddling infants during feeding and only using water as a drink if baby wakes during sleeping; use of cup; only having 2-3 snacks/day; limiting sugar exposure from snacking/sipping.	self-selected by the mother, and was not guaranteed to address bottle cessation, cup use, or cariogenic behaviours.

BBTD: baby bottle tooth decay; CG: control/comparator group; ECC: early childhood caries; IG: intervention group; mo: HCP: health care professional; month; RCT: randomised controlled trial; SES: socioeconomic status; WIC: Women, Infants and Children program; wks: weeks; UK: United Kingdom; USA: United States of America; yrs: years
