# Supplementary material for: Interventions Targeting Bottle and Formula Feeding in the Prevention and Treatment of Early Childhood Caries, Overweight and Obesity: An Integrative Review
Source: Int J Environ Res Public Health. 2021 Nov 23;18(23):12304. doi: 10.3390/ijerph182312304 (PMC8656950; doi:10.3390/ijerph182312304)
Supplement: Supplementary file 1 [file ijerph-18-12304-s001.zip › Suppt File 6. MMAT.pdf]

Supplement File 6. Mixed Methods Appraisal Tool assessment of included studies (N = 27 studies, 31 references).

A

	Randomised appropriately performed	Groups comparable at baseline	Complete outcome data	Outcome assessors blinded to allocation	Adherence to assigned intervention
Bonuck 2014	−	−	−	−	?
Boonrusmee 2021	−	+	+	+	−
Davies 2005	−	−	−	+	?
Franco 2008	−	+	−	+	?
Joury 2016	+	−	+	−	?
Karasz 2018	+	+	+	+	?
Kavanagh 2008	−	+	−	+	?
Kowash 2000	−	+	−	−	?
Lakshman 2018	+	+	+	+	+
Maguire 2010	+	−	+	+	?
Smithers 2017, Jamieson 2018, Jamieson 2019	+	+	−	+	?
Ventura 2021	+	+	+	−	?
Weber-Gasparoni 2013a and 2013b	+	−	−	+	?

B

	Participants representative of the target population	Appropriate measurements for the outcome and intervention	Complete outcome data	Confounders accounted in design and analysis	Adherence to assigned intervention
Borghese-Lang 2003	?	+	−	−	+
Braun 2017	+	+	?	+	+
Bruerd 1989	+	+	?	−	?
Bruerd 1996	+	+	?	−	+
Cheng 2019	+	+	+	+	?
Hamilton 1999	+	+	−	−	+
Harrison 2003	?	+	−	−	?
Hoeft 2016	+	+	−	+	+
Kahn 2007	+	+	+	−	?
Koelen 2000	?	+	+	−	?
Lawrence 2004	+	+	−	−	+
Schroth 2015	+	+	?	?	+
Sgan-Cohen 2001	+	+	+	+	?
Strippel 2010	+	+	+	−	−
Vichayanrat 2012	+	?	−	−	−

A: quantitative randomised controlled trial design. B: quantitative non-randomised study design.
